# Screening Campaign and Docking Investigations in Identifying New Hit Compounds as Inhibitors of Human Carbonic Anhydrases Expressed In Tumour Cells

**DOI:** 10.1002/cmdc.202300330

**Published:** 2023-09-22

**Authors:** Federico Ricci, Andrea Angeli, Francesca Mancuso, Laura De Luca, Claudiu T. Supuran, Rosaria Gitto

**Affiliations:** ^1^ CHIBIOFARAM Department University of Messina Viale F. d'Alcontres 31 98166 Messina Italy; ^2^ NEUROFARBA Department University of Florence Via Ugo Schiff 6 50019 Sesto Fiorentino Italy

**Keywords:** antitumor agents, arylsulfonamides, carbonic anhydrase, docking studies, molecular modeling

## Abstract

The tumor‐expressed human carbonic anhydrase (hCA) isoforms hCA IX and hCA XII have been extensively studied to develop anticancer agents targeting solid tumors in combined therapy. These CA  isoforms are considered key factors in controlling tumor microenvironment (TME) of cancer lines that develop high metastatic activity. Herein, we report the discovery of potent hCA IX/hCA XII inhibitors that were disclosed through a screening campaign on an in‐house collection of arylsulfonamides preliminary tested toward other hCAs. Among them, the *N*‐(4‐sulfamoylphenyl)naphthalene‐2‐carboxamide (**12**) and *N*‐(4‐sulfamoylphenyl)‐3,4‐dihydroisoquinoline‐2(1*H*)‐carbothioamide (**15**) proved to be the most intriguing hCA IX/hCA XII inhibitors displaying favourable selectivity ratios over widespread hCA I and hCA II isoforms. To explore their binding mode, we conducted docking studies that described the poses of the best inhibitors in the catalytic site of hCA IX and hCA XII, thus suggesting the privileged pattern of interactions. These structural findings might further improve the knowledge for a successful identification of new sulfonamides as adjuvant agents in cancer management.

## Graphical abstract

We explored our in‐house collection of sulfonamides to identify new potent hCA IX/XII inhibitors. Docking simulations highlighted the docking poses in catalytic sites of hCA IX and hCA XII cavities. These structural findings may help lead to the successful identification of new sulfonamides as adjuvant agents in cancer management.

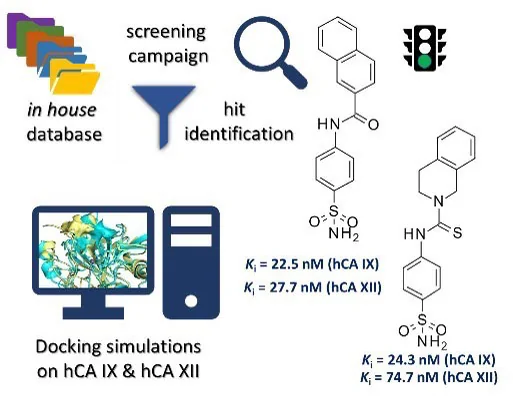

## Introduction

Carbonic Anhydrases (CAs) are metalloenzymes catalysing the very simple hydration reaction providing bicarbonate and proton from carbon dioxide in various biological systems. CAs are expressed in several organisms such as animals, bacteria, fungi, and so on. In humans, the alpha‐class of CAs exerts a fine tuning in CO_2_ concentration thus controlling several physiological processes. It has been well‐established that human CAs (hCAs) play a significant role also in distinct pathological pathways, for which the pH balance is related to cellular signalling and/or protein expression. There are fifteen hCAs (hCA I–XIV, with a pair of the mitochondrial hCA V isoforms VA and VB) having different tissue and cellular distribution. They possess a very different catalytic efficiency and involvement in various pathologies, so that the therapeutic applications of hCA inhibitors (hCAIs) is challenging toward the identification of selective inhibitors targeting specific isoforms.^[1]^ The structural information revealed that a certain homology is shared by the catalytically active CAs, that are generally inhibited by small molecules bearing sulfonamide functional group able to coordinate the zinc ion located to the deep cavity surrounded by three histidine residues.[^2^, ^3^] The above‐mentioned zinc binding group (ZBG) is necessary to anchor the catalytic site; in addition, the most popular hCAIs establish interactions with hydrophobic/hydrophilic residues paving the wall of the cavity and/or specific sub‐pockets.[^4^, ^5^] Particularly, the selectivity profile is optimized when CA Inhibitors are able to show low affinity towards ubiquitous isoforms such as hCA I and hCA II. Searching new theranostic agents in human cancer, recent efforts have been addressed towards hCA IX and hCA XII isoforms, that are overexpressed in cancer cells and are considered responsible in regulating the tumor microenvironment through their influence on extracellular pH.[^6^, ^7^] The hCA IX and hCA XII isozymes are transmembrane proteins having the catalytic site exposed on outside space. A structure‐guided design of hCA IX/XII selective inhibitors is currently focused in the development of CAIs bearing the ZBG moiety linked to suitable single or multiple tails that interact with specific regions characterizing the tumour expressed isoforms.[^8^, ^9^, ^10^] The hCA IX/hCA XII selectivity has been achieved with effective inhibitors bearing ureidobenzenesulfonamide scaffold. Among them, the compound named SLC‐0111 completed Phase II clinical trials for the treatment of hypoxic tumours and as adjuvant agent in advanced solid tumours. Moreover, other studies identified further benzenesulfonamides possessing high hCA IX/hCA XII and improvement isoform selectivity.[^11^, ^12^, ^13^]

Apart from the common zinc binding site (H94, H96, H119, T199), for the different hCAs the active site is well characterized by two distinct areas opposed each other controlling the enrolment of CO_2_ and subsequent release of HCO_3_^−^. In more detail, the hCA IX is schematically composed of two halves: (i) the hydrophobic area is essentially lined by L91, V131, L135 and P202 residues; (ii) the hydrophilic area includes R60, R62, H64, Q67, T69, E170. For hCA XII the presence of (i) one alanine residue at 131 position and (ii) two polar uncharged serine residues at 132 and 135 positions results in the variation of shape and polarity at the entrance of the cavity, thus producing a different pattern of interactions for inhibitors able to discriminate these two tumor expressed isoforms. Moreover, Q67, T69 and L91 of hCA IX are replaced by L61, N69 and T91 in hCA XII. Overall, the cleft of hCA IX and hCA XII displays some features that enable the identification of selective inhibitors for therapeutic purpose.^[14]^ In the present work, we wondered about the possibility of identifying new chemotypes of hCAIs through a screening campaign of a collections of various arylsulfonamides. The best compounds were analyzed for their proposed binding poses within hCA IX and hCA XII cavities.

## Results and Discussion

To address the challenge of identifying novel hit compounds targeting hCA IX and hCA XII, we conducted the biological screening on our small series of benzensulfonamide compounds (**1**–**31**) having acceptable different structural motifs linked to ZBG as displayed in Figures 1 and 2. In details, all tested compounds possess the arylsulfonamide anchoring moiety linked with various hydrophobic rings or polar moieties; the linking group possesses various length and polarity on the basis of the number of hydrogen bond donor and acceptor functionalities. These compounds belong to a series of molecules that we have already tested toward different druggable CA isoforms in our previous papers.[^15^, ^16^]

**Figure 1:**
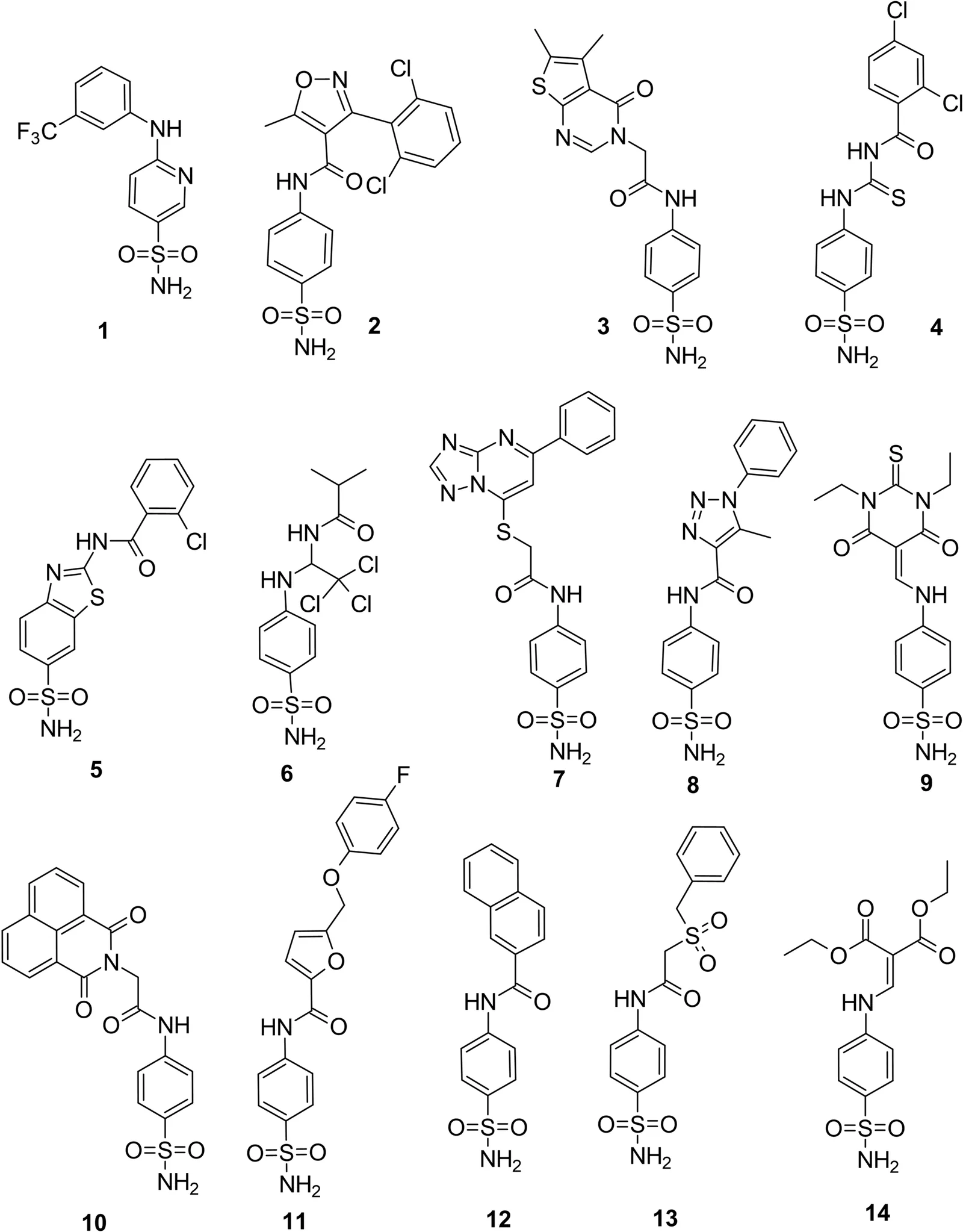
Structures of studied sulfonamides **1**–**14**.

**Figure 2:**
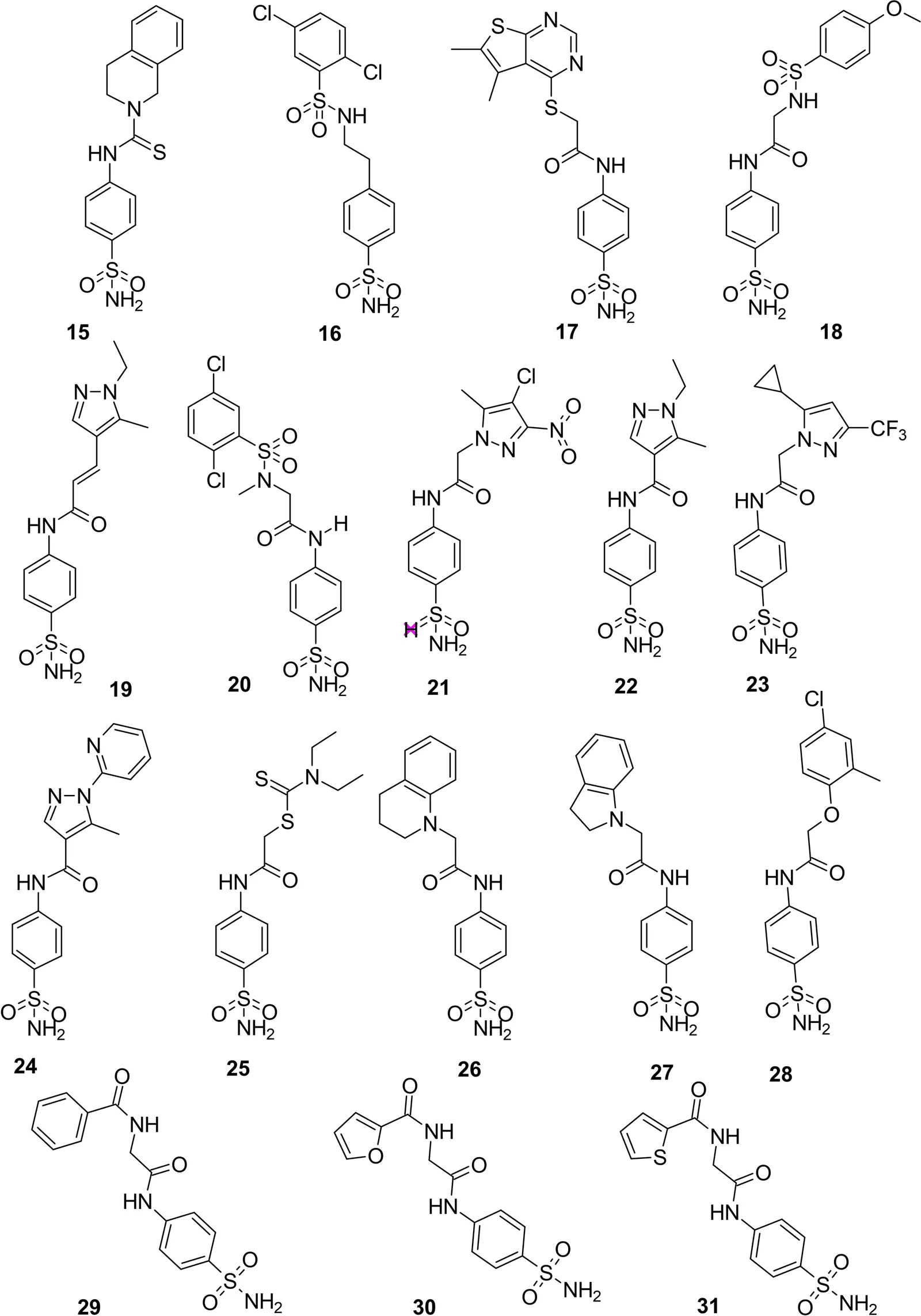
Structures of studied sulfonamides **15**–**31**.

### Assessment of CA Inhibitory assay toward hCA I, hCA II, hCA IX, and hCA XII isoforms

Compounds **1**–**31** were *in vitro* tested using a stopped‐flow carbon dioxide hydrase assay. Table 1 collects their *K*_i_ values against hCA IX and hCA XII, in comparison with ubiquitous hCA I and hCA II and with well‐known CA Inhibitors compounds acetazolamide (AAZ) and SLC‐0111 that were previously tested in the same experimental conditions.[^11^, ^17^]

**Table 1:** *K*_i_ values against hCA I, hCA II, hCA IX, and hCA XII showed by sulfonamide derivatives **1**–**31** and reference compounds AAZ and SLC‐0111.

	Ki [nM]^[a]^					Selectivity ratios^[b]^			
Cmpd.	hCA I	hCA II	hCA IX	hCA XII		hCA I/ hCA IX	hCA II/ hCA IX	hCA I/ hCA XII	hCA II/ hCA XII
**1**	15.9	8.4	22.2	9.5		0.7	0.4	1.7	0.9
**2**	28.7	20	436.5	50.0		0.1	0.05	0.6	0.4
**3**	34.6	8.2	24.5	400		1.4	0.3	0.1	0.02
**4**	23.9	3.7	212.5	57.9		0.1	0.02	0.4	0.1
**5**	39.4	6	172.5	396.3		0.2	0.03	0.1	0.02
**6**	45.3	26.2	184.5	243		0.2	0.1	0.2	0.1
**7**	73.1	14.3	217.5	295.9		0.3	0.1	0.2	0.05
**8**	279.3	23.3	225.3	50.6		1.2	0.1	5.5	0.5
**9**	435.7	46.8	464	314.6		0.9	0.1	1.4	0.1
**10**	9.1	28.2	20.7	8.3		0.4	1.4	1.1	3.4
**11**	92.4	24.2	22.1	38.4		4.2	1.1	2.4	0.6
**12**	954	309.8	22.5	27.7		42.4	13.8	34.4	11.2
**13**	72	28.2	22.1	9.2		3.3	1.3	7.8	3.1
**14**	18.2	5.1	22.2	62.6		0.8	0.2	0.3	0.1
**15**	411.2	658.2	24.3	74.7		16.9	27.1	5.5	8.8
**16**	4.6	3.8	23.5	87.9		0.2	0.2	0.1	0.04
**17**	927.3	311.2	88.1	58.5		10.5	3.5	15.9	5.3
**18**	47.9	7.4	20.9	32.1		2.3	0.4	1.5	0.2
**19**	53.4	18.2	23.5	73.3		2.3	0.8	0.7	0.2
**20**	67.9	20.9	209.7	66.4		0.3	0.1	1.0	0.3
**21**	77.9	22.4	107.4	64.3		0.7	0.2	1.2	0.3
**22**	27.1	5.6	165.6	8.8		0.2	0.03	3.1	0.6
**23**	57.4	9.2	146.3	87.8		0.4	0.1	0.7	0.1
**24**	86.3	13.6	168.5	55.8		0.5	0.1	1.5	0.2
**25**	52.5	18.9	128.6	97.4		0.4	0.1	0.5	0.2
**26**	927.4	379.1	210.3	82.5		4.4	1.8	11.2	4.6
**27**	76.9	26.3	200.6	182.6		0.4	0.1	0.4	0.1
**28**	60.1	21.7	50.6	82.5		1.2	0.4	0.7	0.3
**29**	524.3	19.9	204.4	39.2		2.6	0.1	13.4	0.5
**30**	429.6	38.5	239.1	253.9		1.8	0.2	1.7	0.2
**31**	620.8	22.2	193.7	90.4		3.2	0.1	6.9	0.2
**SLC‐0111**	5080	960	45	4.5		112.9	21.3	1128.9	213.3
**AAZ**	250	12.1	25.7	5.7		9.7	0.5	43.9	2.1

[a] Errors in the range of ±5–10 % of the reported values, from 3 different assays. [b] Selectivity as determined by the ratio of *K*_i_s for hCA I or hCA II relative to hCA IX or hCA XII.

Because the arylsulfonamide moiety of compounds **1**–**31** is not linked to a homogenous series of chemical tails, a canonical structure‐affinity relationship analysis is hampered. Therefore, the discussion on the results reported on Table 1 was exclusively focused on the identification of a good balance of inhibitory effects toward antitumor hCA IX/XII isoforms and significant selectivity over hCA I and hCA II as measured by the determination of selectivity ratios. By analyzing the data shown in Table 1, we can observe that the eleven sulfonamide compounds **1**, **3**, **10**, **11**, **12**, **13**, **14**, **15**, **16, 18, 19** proved to be the best active hCA IX inhibitors with *K*_i_ values falling in a close range from 20.7 to 24.5 nM, so that they resulted more efficacious inhibitors when compared to SLC‐0111 (*K*_i_ value of 45 nM). Among them, compounds **12** and **15** demonstrated nice selectivity over hCA I and hCA II. In more detail, the *N*‐(4‐sulfamoylphenyl)naphthalene‐2‐carboxamide (**12**) was about 42‐fold more active against hCA IX over hCA I. Noteworthily, the *N*‐(4‐sulfamoylphenyl)‐3,4‐dihydroisoquinoline‐2(1*H*)‐carbothioamide (**15**) was about 27‐fold more active against hCA IX over hCA II, thus demonstrating a selectivity profile that resulted very similar to that of the active agent SLC‐0111 (about 21‐fold selective against hCA IX over hCA II).

Concerning the inhibitory effects toward hCA XII, our study revealed that the four sulfonamides **1**, **10**, **13**, and **22** demonstrated very high activity against this isoform possessing *K*_i_ values in low nanomolar range (from 8.3 to 9.5 nM); these values were very close to the that of well‐known inhibitor SLC‐0111; unfortunately, these potent hCAX II inhibitors failed to demonstrate high selectivity when compared to SLC‐0111 (see Table 1).

Interestingly, the sulfonamide **12** proved to be medium weaker inhibitor of hCA XII (*K*_i_ value of 27.7 nM) to respect the above‐mentioned sulfonamides **1**, **10**, **13**, and **22**; however, inhibitor **12** demonstrated the most promising selectivity over hCA I and hCA II. Overall, our studies indicated that the sulfonamide *N*‐(4‐sulfamoylphenyl)naphthalene‐2‐carboxamide (**12**) was the most efficient and selective inhibitor of tumor‐expressed hCA IX and hCA XII isoform.

We selected compounds **12**, **15**, **26**, **29**, and **31** for a preliminary evaluation of the viability toward selected human breast carcinoma (MCF‐7) and human colon rectal carcinoma (HCT‐116) cell lines by using the CCK‐8 method under normoxic cell cancer conditions. Overall, these compounds were poor inhibitors of cell proliferation under the fixed doses of 10 μM for each compound. In particular, the screening revealed that the best percentage of inhibition rate in MCF‐7 cell was measured for inhibitors **15** (8.45 %), **26** (8.36 %), and **31** (8.65 %); whereas only compound **12** demonstrated to affect cell viability of HTC‐116 cell lines displaying a poor percentage of inhibition of 1.82 %. These data suggested that for this class of arylsulfonamides there is not a clear correlation between the inhibition of hCA IX and hCA XII isoforms and activity in MCF‐7 and HCT‐116 cell lines under standard conditions of cellular proliferation.

### Inhibitor binding analysis in hCA IX and hCA XII catalytic sites

Computational studies were performed to predict the binding mode of compounds **12** and **15** within hCA IX and hCA XII as prototypes of two arylsulfonamides combining affinity and selectivity towards the above‐mentioned isoforms. We applied the same docking protocols that we have employed in previous papers to analyze the binding poses of inhibitors targeting hCA IX/XII.[^5^, ^18^] The two inhibitors **12** (magenta, Figure 3A) and **15** (green, Figure 3B) displayed an identical binding mode on hCA IX; they formed the canonical polar interaction with T199 nitrogen as well as coordinated the zinc ion through their ZBG (Figure 3C). These findings were in good agreement with the biochemical data displayed in Table 1. As displayed in Figure 3, both ligands anchored the binding pocket through a crucial hydrogen bond with Q92 and formed several lipophilic interactions at the entrance of the binding site, justifying the low nanomolar affinity toward the enzyme. In detail, the naphthalene and isoquinoline tails were projected toward the of the protein cavity maintaining hydrophobic contacts with V131 and L135.

**Figure 3:**
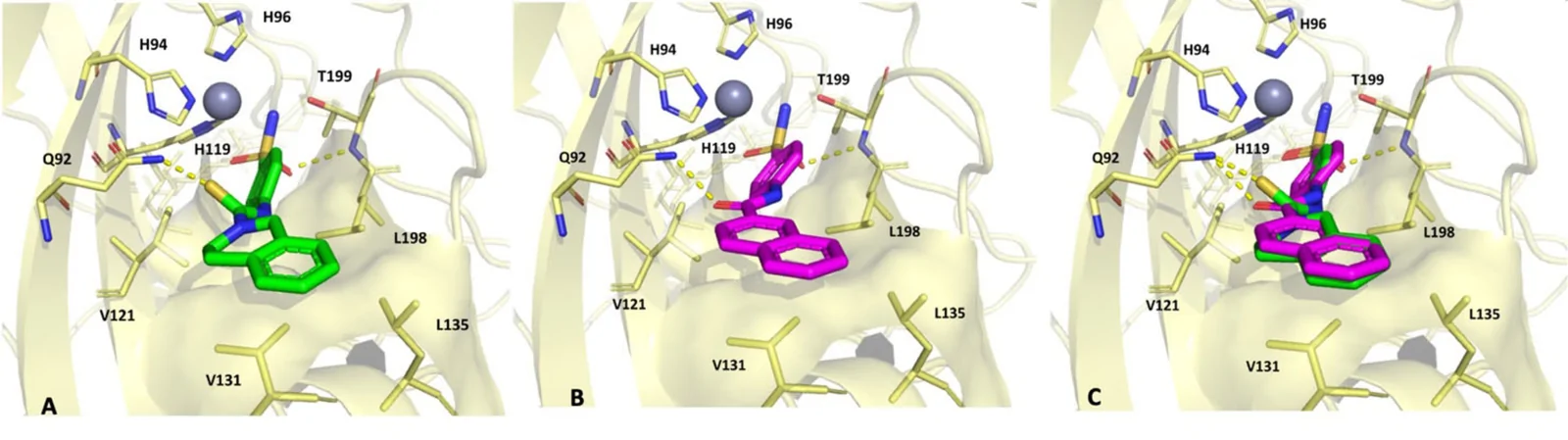
Suggested binding modes of compounds **12** (magenta, panel A) and **15** (green, panel B) docked into the hCA IX structure (PDB code 3IAI). The key residues of the pocket are presented, and the hydrogen‐bond interactions are shown by dotted lines. Panel C: superimposition of the docking poses of compounds **12** and **15**. The interactions between hCA IX and inhibitors **12** and **15** were examined using PyMOL (https://pymol.org).

As shown in Figure 4 the arylsulfonamide moieties of studied inhibitors **12** (pink, panel A) and **15** (blue‐green, panel B) inserted well into the bottom of hCA XII cavity. In detail, the two aromatic rings bound the zinc ion as well as T199, T200 and L198 residues through polar and hydrophobic interactions. On the contrary, the two hydrophobic tails adopted a very different orientation into the wide entrance of hCA XII cavity (see Figure 4C). The inhibitor **12** (coloured in pink) maintained the hydrophobic interactions with the surrounding residues A131 and T91. For compound **15** (coloured in blue‐green), the docking simulations predicted the isoquinoline tails directed to an opposite area without making significant contacts with hydrophobic residues (Figures 4B and 4C). The difference in the binding mode in hCA XII proposed for the two studied inhibitors suggested that the wide space at the entrance of hCA XII active site may favour bulkier and planar aromatic ring linked to benzene‐sulfonamide moiety (Figure 4C) due to a better shape complementarity. This observation agrees as well with the biological data provided by the two inhibitors (see Table 1).

**Figure 4:**
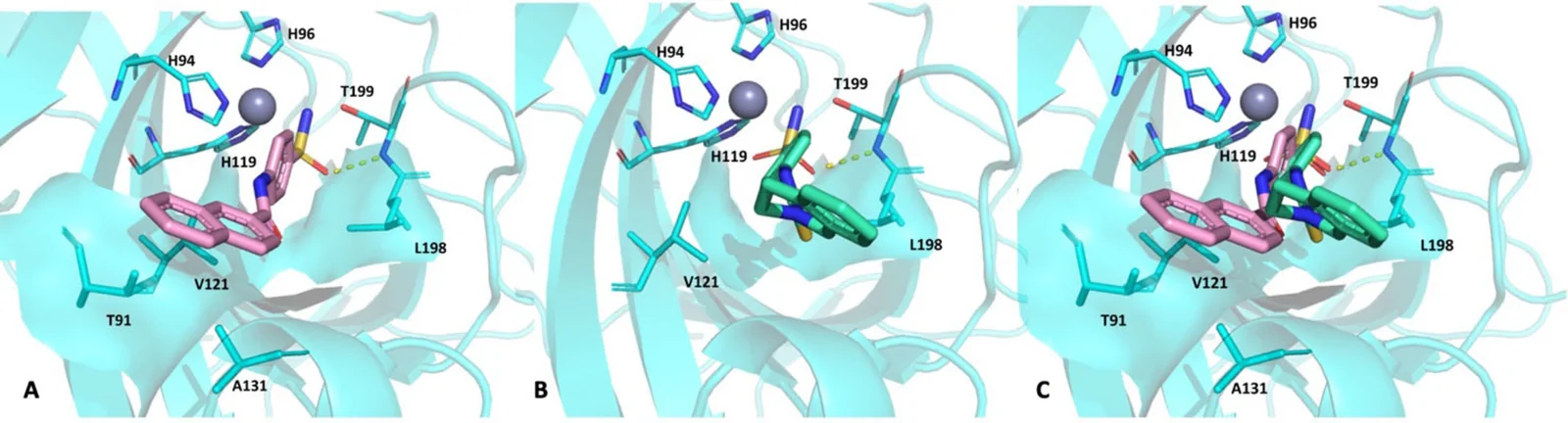
Suggested binding modes of compounds **12** (pink, panel A) and **15** (blue‐green, panel B) docked into the hCA XII structure (PDB code 1JD0). The key residues of the pocket are presented, and the hydrogen‐bond interactions are shown by dotted lines. In panel C a superimposition of docking poses of compounds **12** and **15**. The interactions between hCA XII and **12** and **15** were examined using PyMOL (https://pymol.org).

## Conclusions

A collection of various sulfonamides was *in vitro* screened as candidate inhibitors against hCA IX/XII isoforms; to assess their selectivity we compared their *K*_i_ values with affinity toward the two widespread CA Isoforms hCA I and hCA II. The screening campaign allowed us to identify new promising inhibitors **12** and **15**, for which the docked poses were suggestive of the role of aromatic tail in occupying the middle area of CA cavity. These preliminary achievements might drive a further designing of new analog compounds possessing improved affinity combined with cellular efficacious.

## Experimental Section

### Chemistry

The thirty‐one sulfonamides **1**–**31** are derived from distinct sources. The twenty‐eight sulfonamide derivatives **1**–**28** were bought from Specs (http://www.specs.net/) and used without purification. In details, the Specs database is a collection of commercially available compounds for purchasing and testing. The purity of the compound reported by the manufacturer was greater than 95 %. For several selected and un‐characterized compounds retrieved from Specs database, the ^1^H‐NMR data were collected and are reported in Supporting Information (Figures S1–S20). The compounds **29**–**31** were reported in our previous paper in which the synthetic route and structural characterization were carefully described.^[16]^

### CA Inhibition measurements

The CA Inhibition has been measured by a stopped‐flow method monitoring CO_2_ hydration, as reported earlier.[^11^, ^17^]

### Cellular viability on MCF‐7 and HCT‐116 cell lines

The cytotoxicity of the selected compounds on the human colorectal cell line (HCT‐116) and human breast cell line (MCF‐7) was evaluated by CCK‐8 method. The assay was performed through the cell‐based screening platform of MedChemExpress Ltd. The tested compound was applied at fixed concentration of 10 μM; each concentration was repeated three times, the compound treatment time was 48 hrs. The detailed protocol is available on http://www.medchemexpress.com/.

### Docking protocol for hCA IX‐mimic and hCA XII

Ligands were prepared for docking using VEGA ZZ (3.2.2):^[19]^ 2D structures were sketched using Ketcher editor implemented in VEGA ZZ; protonation state of sulfonamide nitrogen was set as deprotonated; charges and potentials were calculated using Gasteiger method and SP4 forcefields; 1000 minimization steps were performed using Ammp, using a toler value of 0.01; conformational search on all the flexible torsions was made using the systematic method on Ammp and all the default settings; the final minimization using the same settings of step 4 was done on the conformation obtained through conformational search, then saved as mol2 file. Crystal structures of hCA IX (PBD code 3IAI) and XII (PDB code 1JD0) in complex with acetazolamide were downloaded from RCSB PDB and then processed on Maestro (version 2020) using the default settings of Protein preparation wizard. For the docking studies were applied the same procedure described in our previous papers[^5^, ^18^] papers for hCA IX and XII were applied on 100 GA runs (deactivating the “allow early termination” option) of GOLD software.^[20]^ In detail, for both hCA IX and XII a 10 Å radius from the co‐crystallized acetazolamide centroid was set. Then, for hCA IX docking simulations the Chemscore scoring function has been employed; while for hCA XII docking studies we used ChemPLP and applied a scaffold constrain (penalty=0.5) to restrict the possible docking solutions to the ones in which the sulfonamide act as ZBG. The top ranked pose of each compound was selected for binding mode analysis in LigandScout.^[21]^

## Supporting Information

The Supporting Information contains ^1^H NMR spectroscopic data for selected compounds.

## Conflict of interest

The authors declare no conflict of interest.

## Supporting information

As a service to our authors and readers, this journal provides supporting information supplied by the authors. Such materials are peer reviewed and may be re‐organized for online delivery, but are not copy‐edited or typeset. Technical support issues arising from supporting information (other than missing files) should be addressed to the authors.

Supporting Information

## Data Availability

The data that support the findings of this study are available in the supplementary material of this article.
