# Supplementary material for: Screening Campaign and Docking Investigations in Identifying New Hit Compounds as Inhibitors of Human Carbonic Anhydrases Expressed In Tumour Cells
Source: ChemMedChem. 2023 Sep 22;18(20):e202300330. doi: 10.1002/cmdc.202300330 (PMC13652704; doi:10.1002/cmdc.202300330)
Supplement: Supplementary file 1 [file CMDC-18-e202300330-s001.pdf]

ChemMedChem

Supporting Information

Screening Campaign and Docking Investigations in Identifying New Hit Compounds as Inhibitors of Human Carbonic Anhydrases Expressed In Tumour Cells

Federico Ricci, Andrea Angeli, Francesca Mancuso, Laura De Luca, Claudiu T. Supuran, and Rosaria Gitto*

Selected representative ^{1}H-NMR spectra of studied compounds

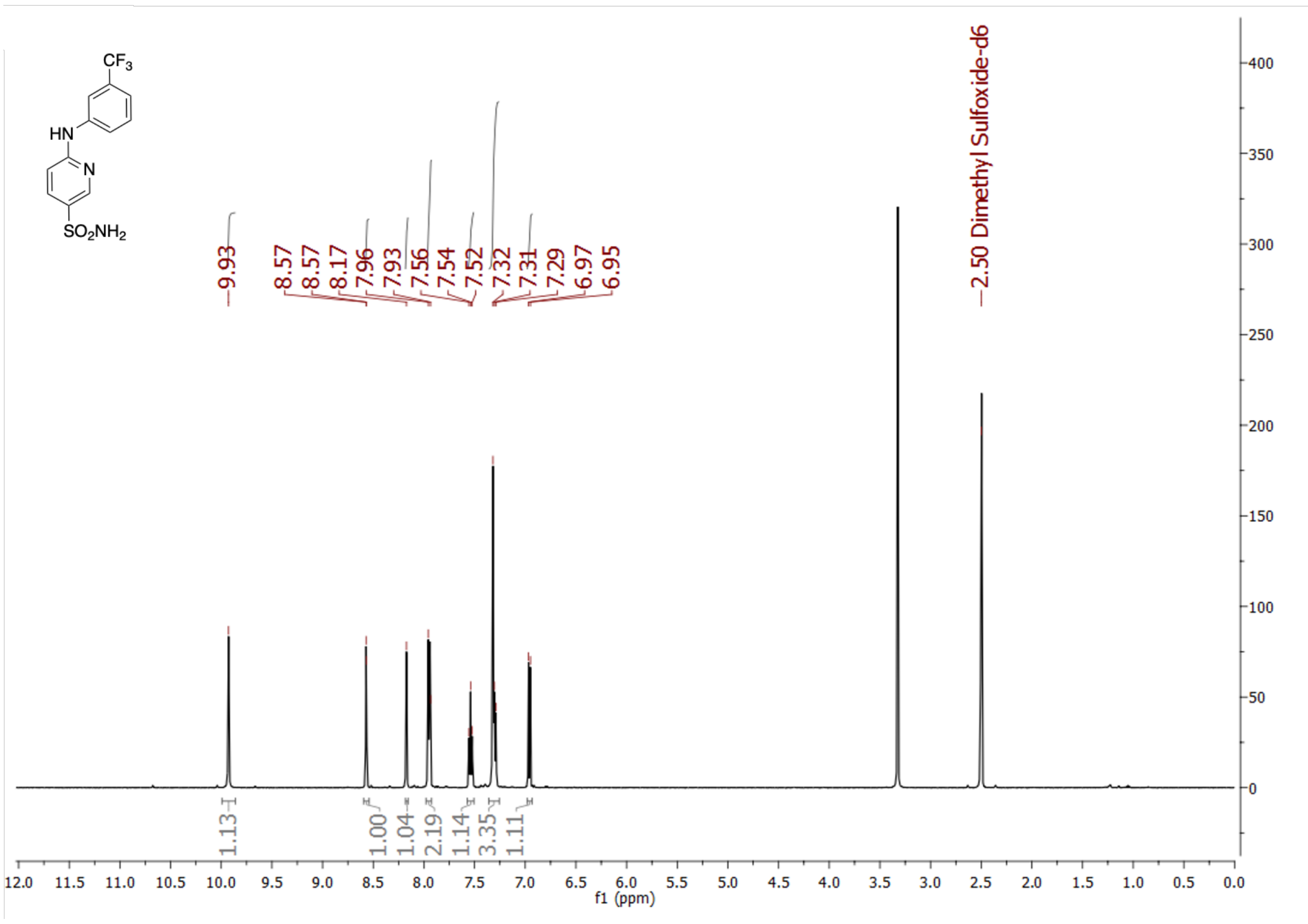

Fig. S1: ^{1}H NMR spectrum of 6-((3-(trifluoromethyl)phenyl)amino)pyridine-3-sulfonamide (**1**)

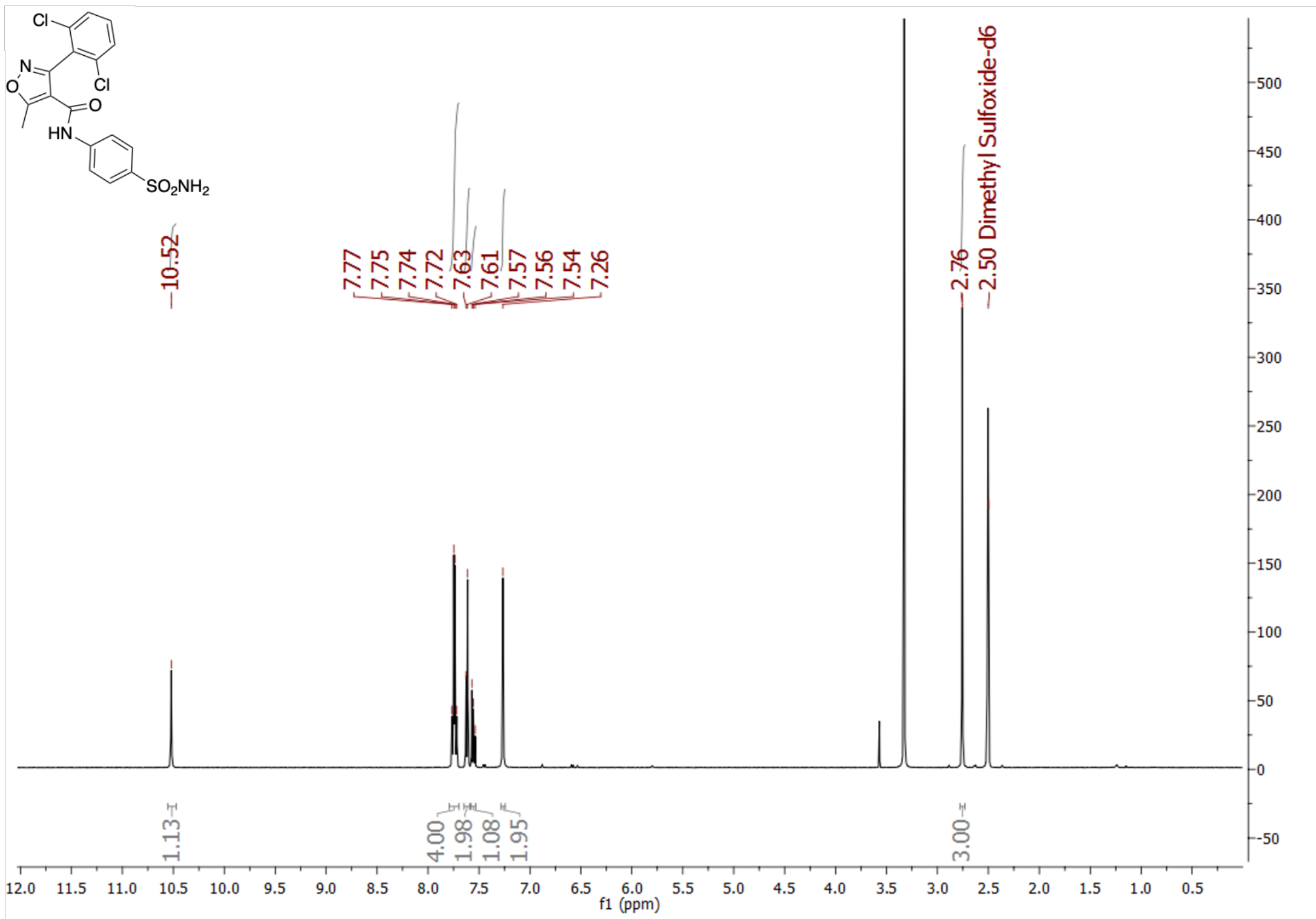

Fig. S2: ^{1}H NMR spectrum of 3-(2,6-dichlorophenyl)-5-methyl-*N*-(4-sulfamoylphenyl)isoxazole-4-carboxamide (**2**)

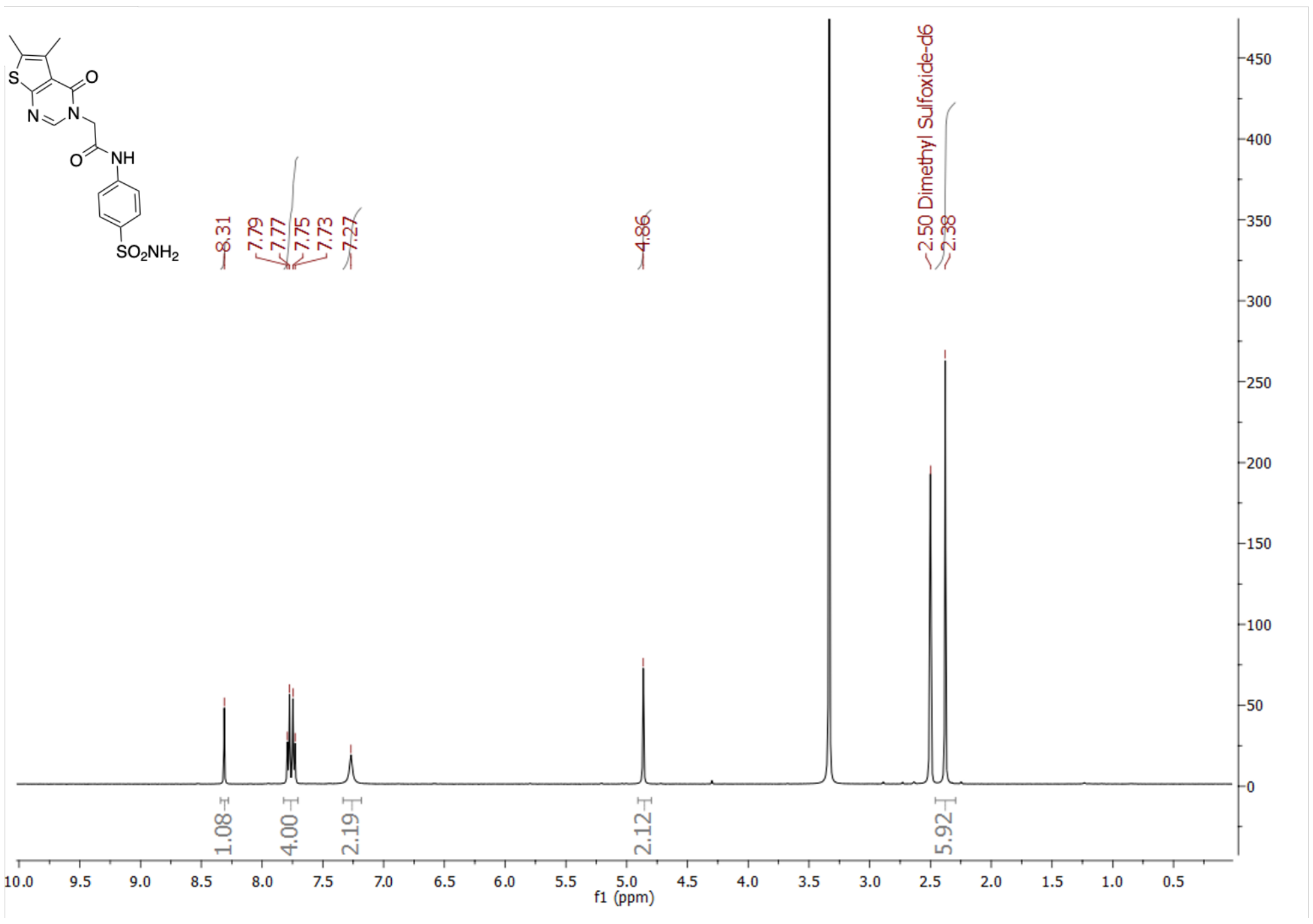

Fig. S3: ^{1}H NMR spectrum of 2-(5,6-dimethyl-4-oxothieno[2,3-*d*]pyrimidin-3(4*H*)-yl)-*N*-(4-sulfamoylphenyl)acetamide (**3**)

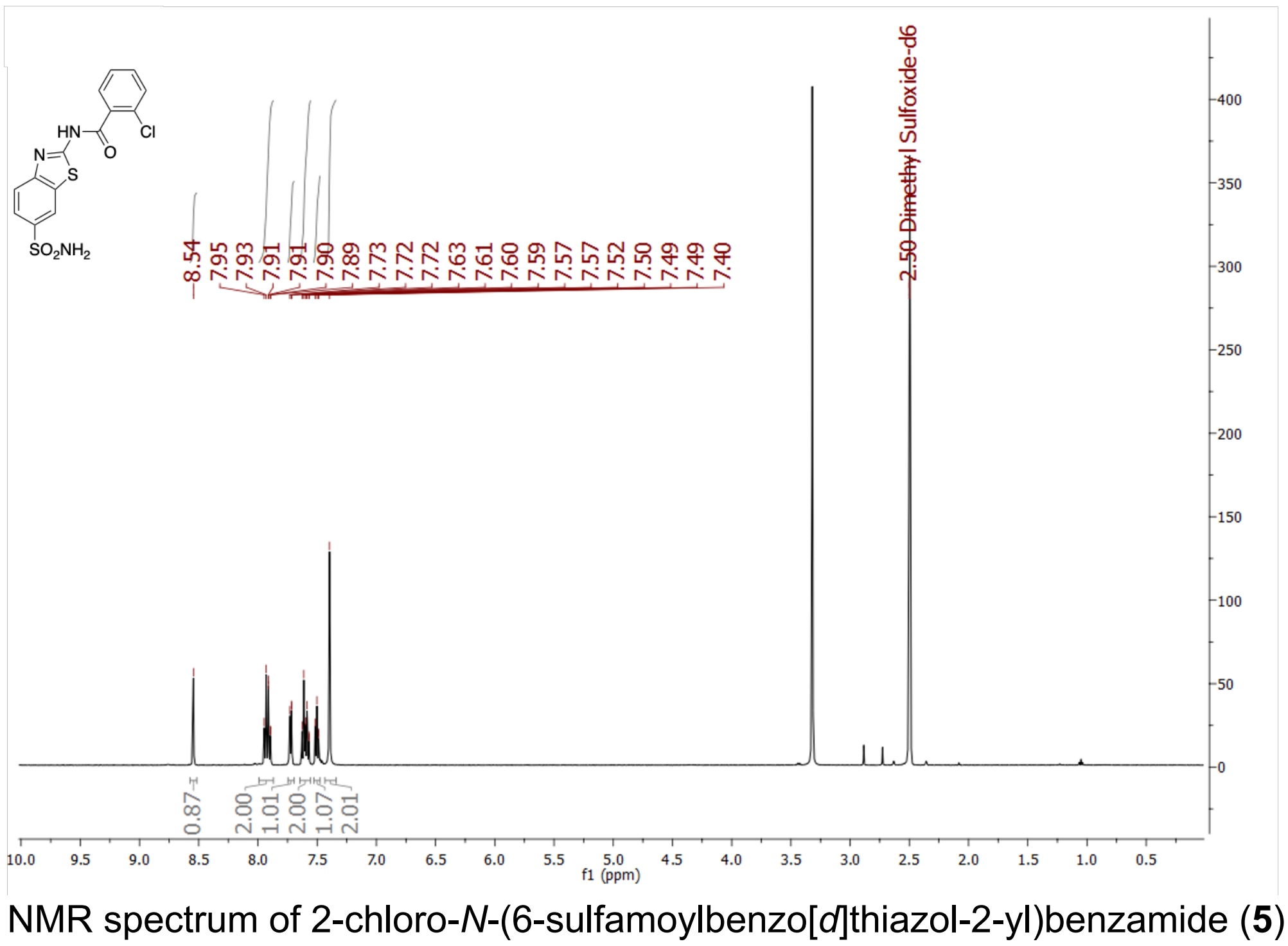

Fig. S4: ^{1}H NMR spectrum of 2-chloro-*N*-(6-sulfamoylbenzo[*d*]thiazol-2-yl)benzamide (**5**)

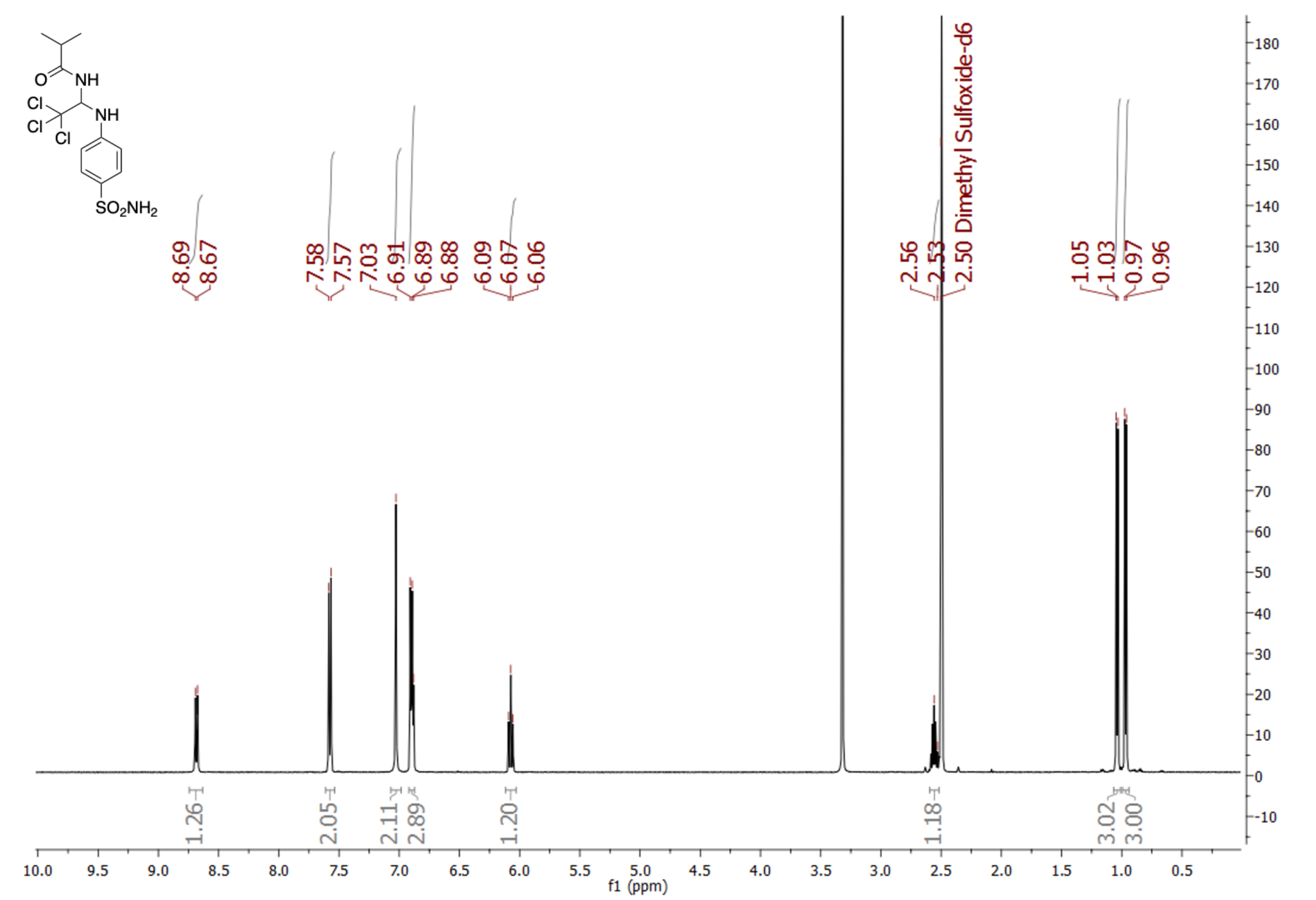

Fig. S5: ^{1}H NMR spectrum of *N*-(2,2,2-trichloro-1-((4-sulfamoylphenyl)amino)ethyl)isobutyramide (**6**)

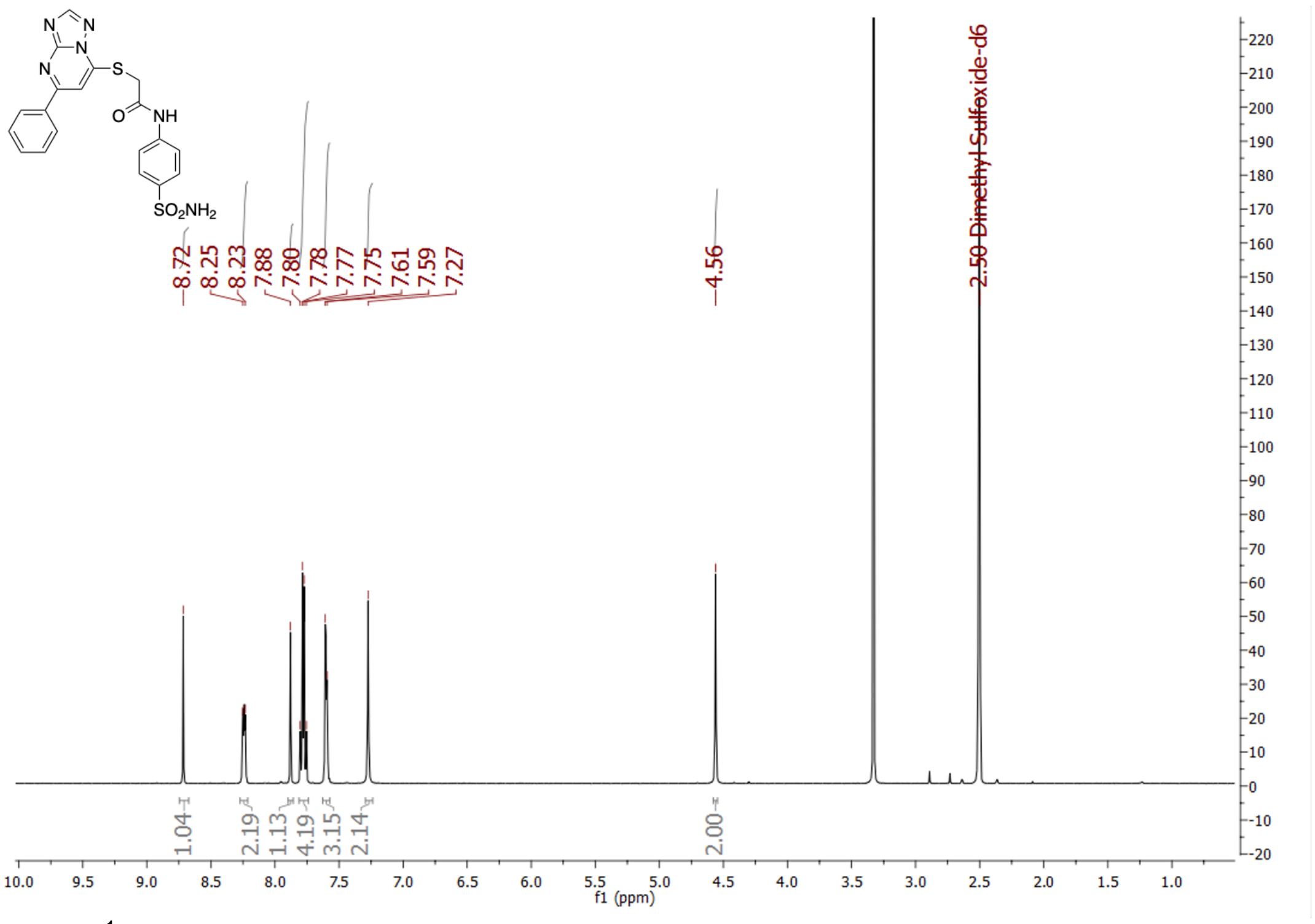

Fig. S6: ^{1}H NMR spectrum of 2-((5-phenyl-[1,2,4]triazolo[1,5-*a*]pyrimidin-7-yl)thio)-*N*-(4 sulfamoylphenyl)acetamide (**7**)

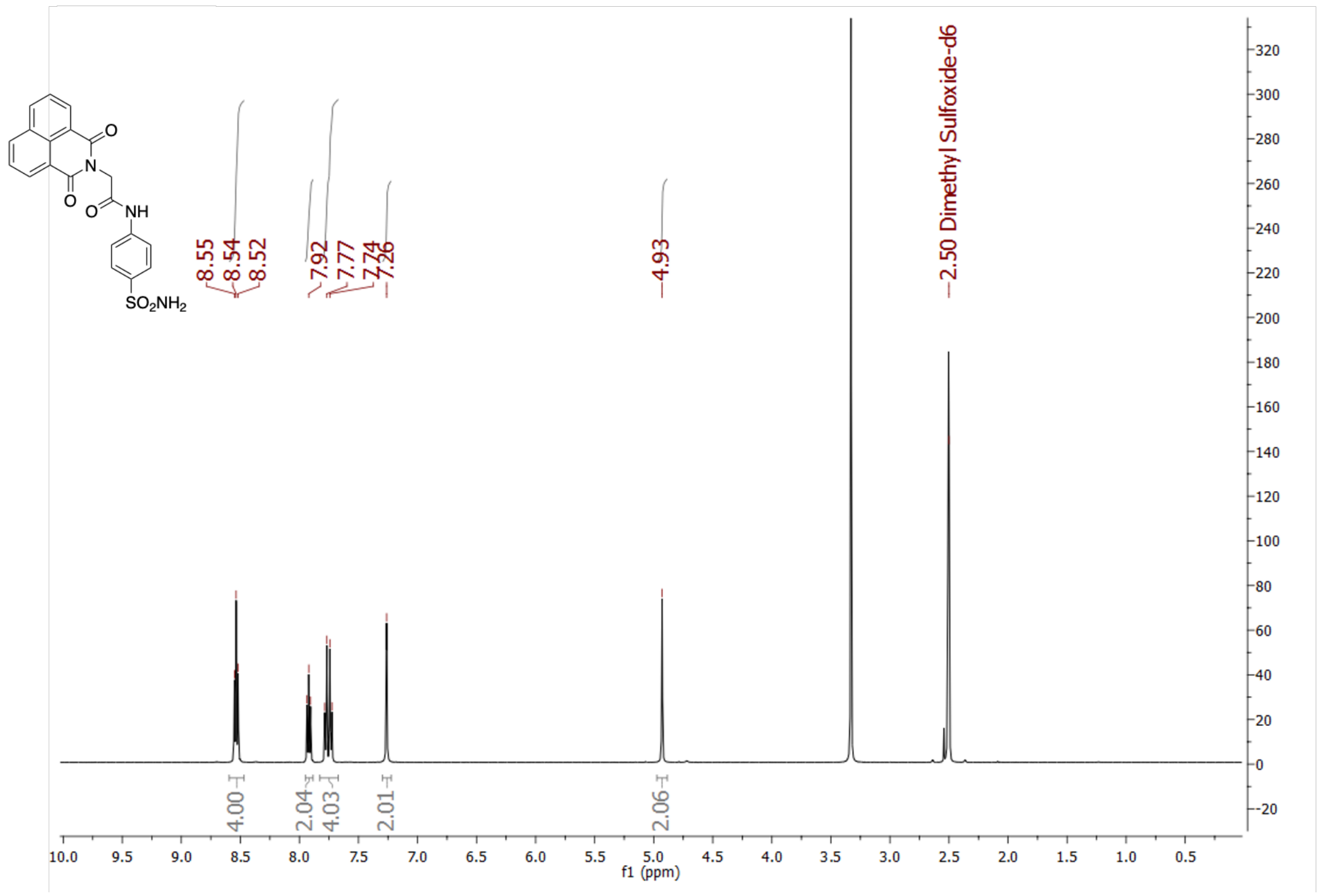

Fig. S7: ^{1}H NMR spectrum of 2-(1,3-dioxo-1*H*-benzo[*de*]isoquinolin-2(3*H*)-yl)-*N*-(4-sulfamoylphenyl)acetamide (**10**)

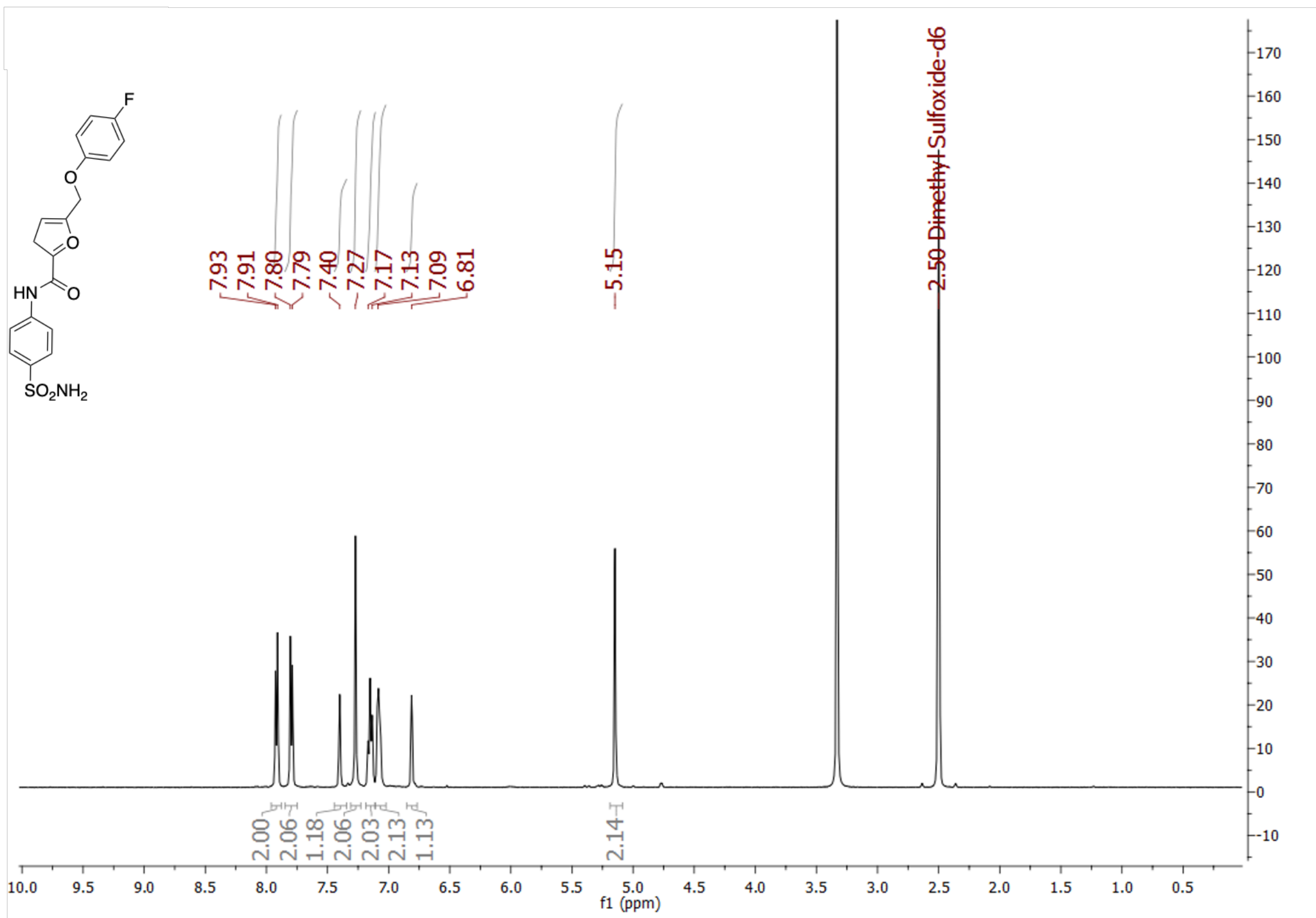

Fig. S8: ^{1}H NMR spectrum of 5-((4-fluorophenoxy)methyl)-*N*-(4-sulfamoylphenyl)-furan-2-carboxamide (**11**)

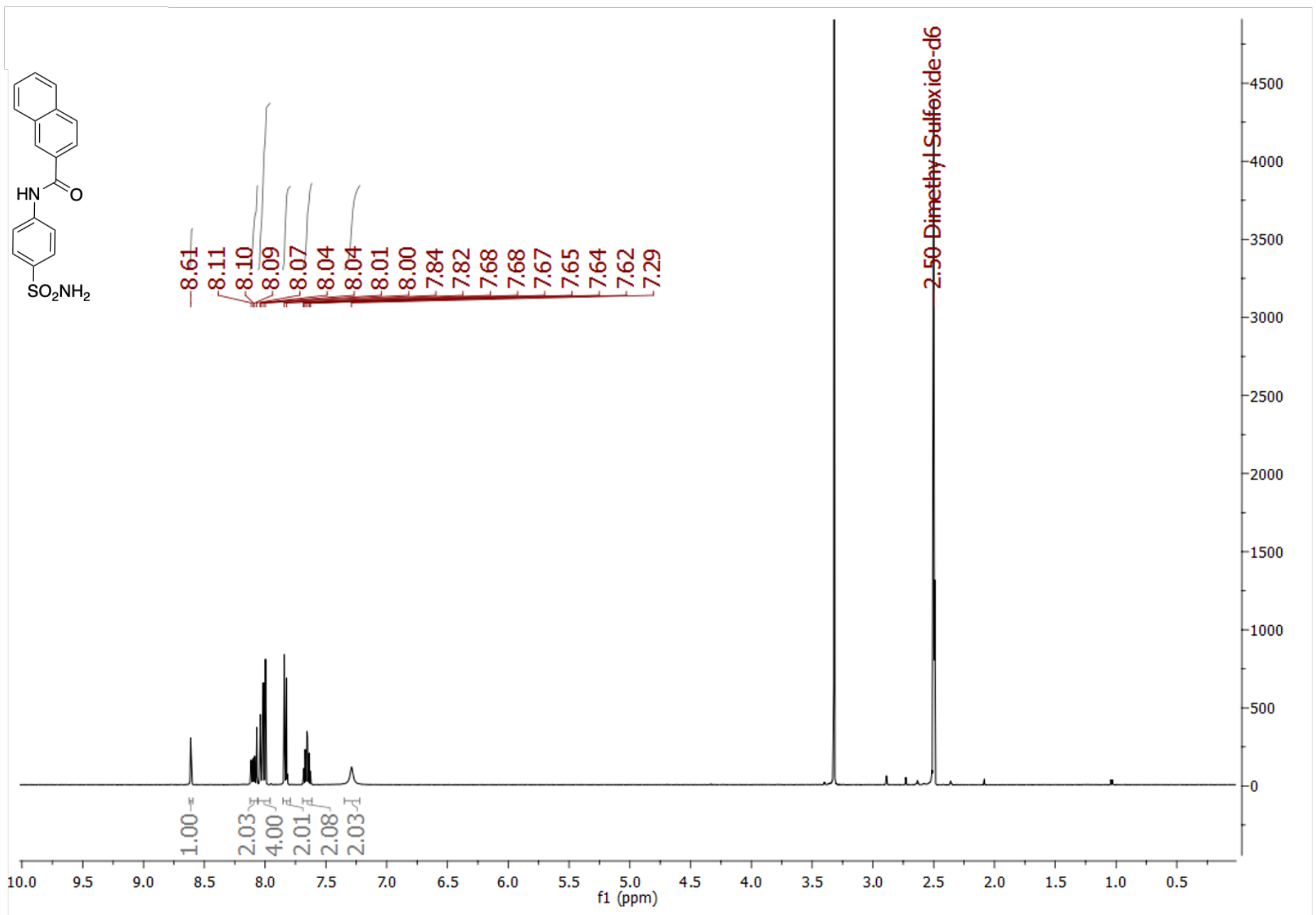

Fig. S9: ^{1}H NMR spectrum of *N*-(4-sulfamoylphenyl)naphthalene-2-carboxamide (**12**)

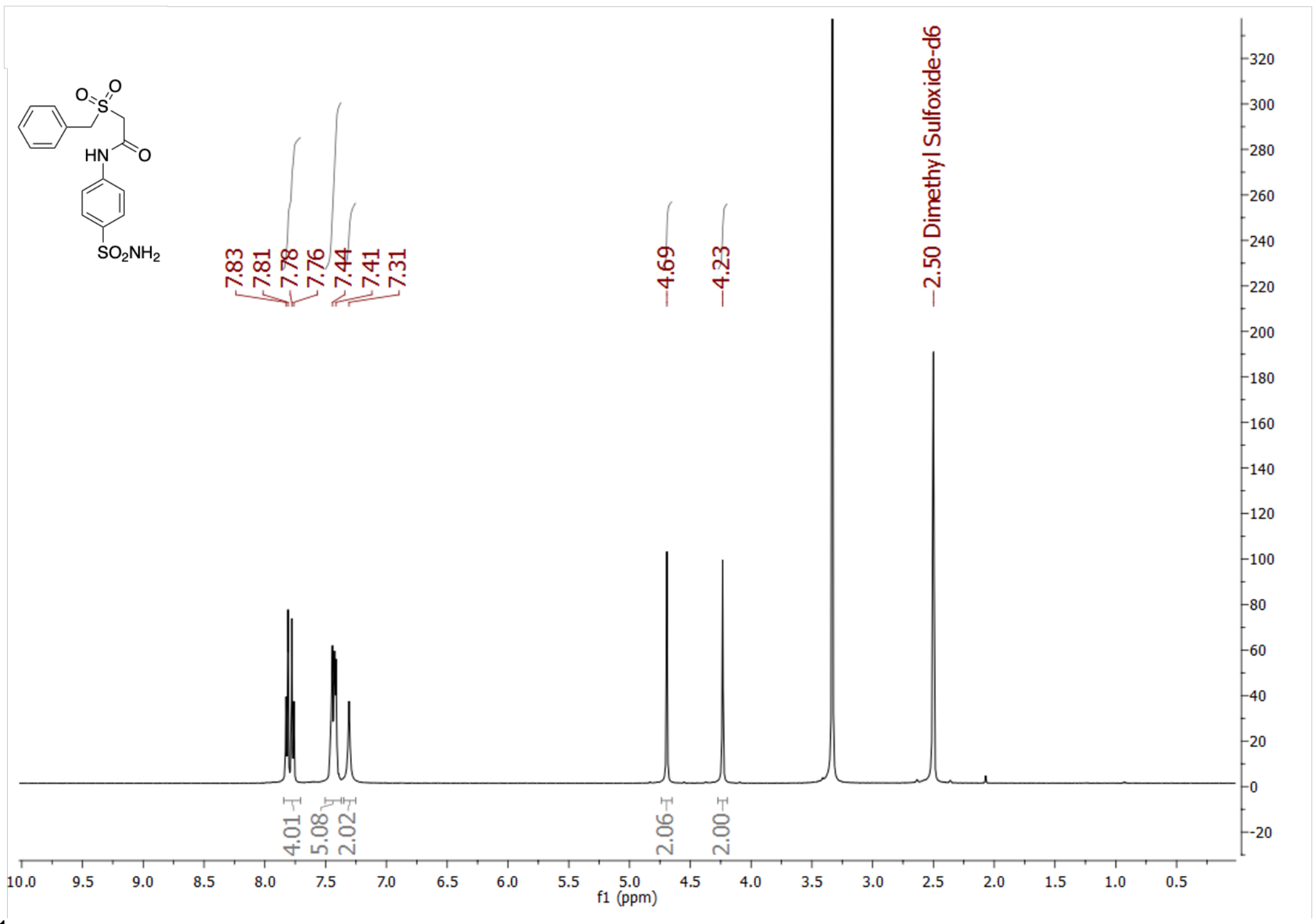

Fig. S10: ^{1}H NMR spectrum of 2-(benzylsulfonyl)-*N*-(4-sulfamoylphenyl)acetamide (**13**)

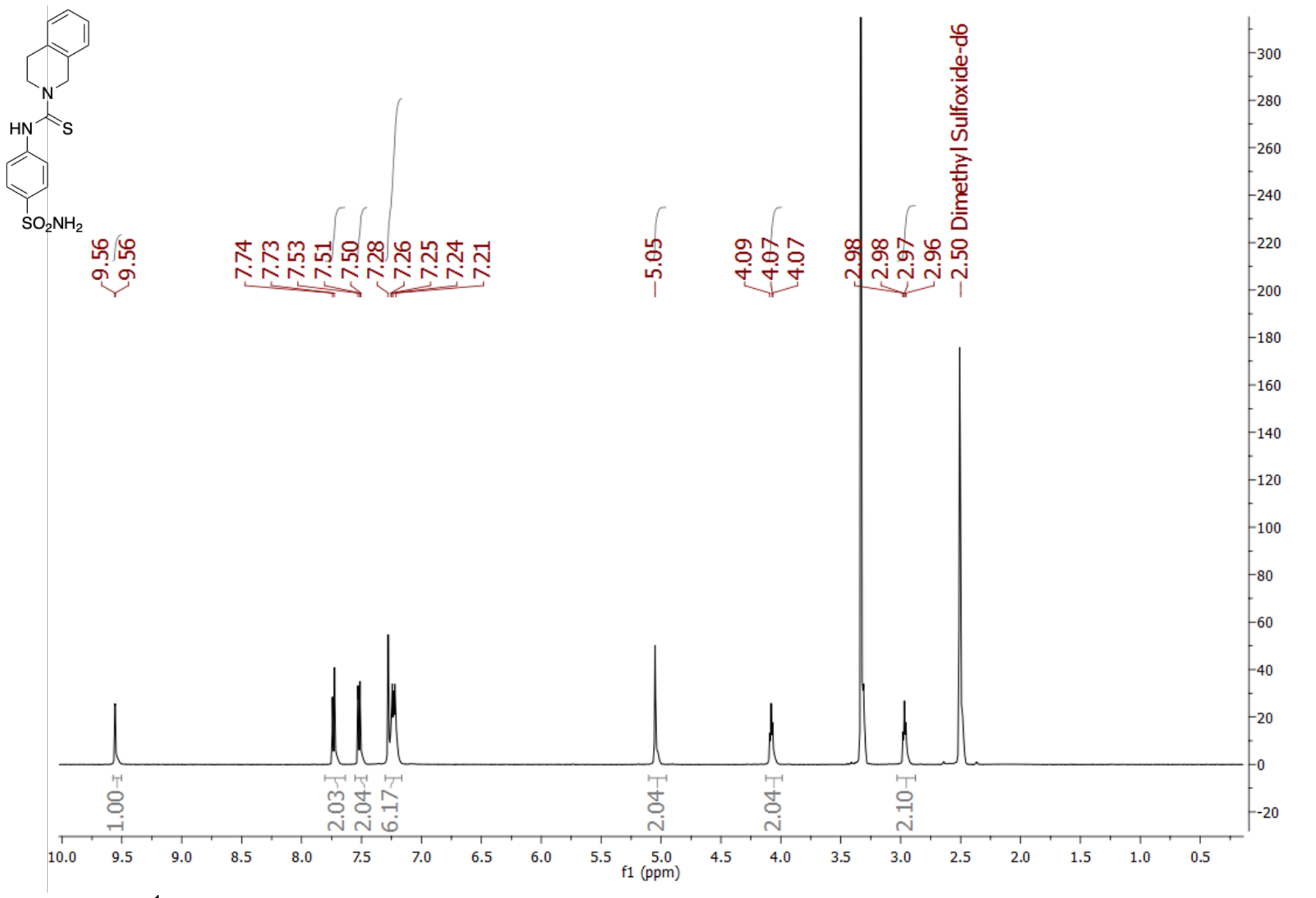

Fig. S11: ^{1}H NMR spectrum of *N*-(4-sulfamoylphenyl)-3,4-dihydroisoquinoline-2(1*H*)-carbothioamide (**15**)

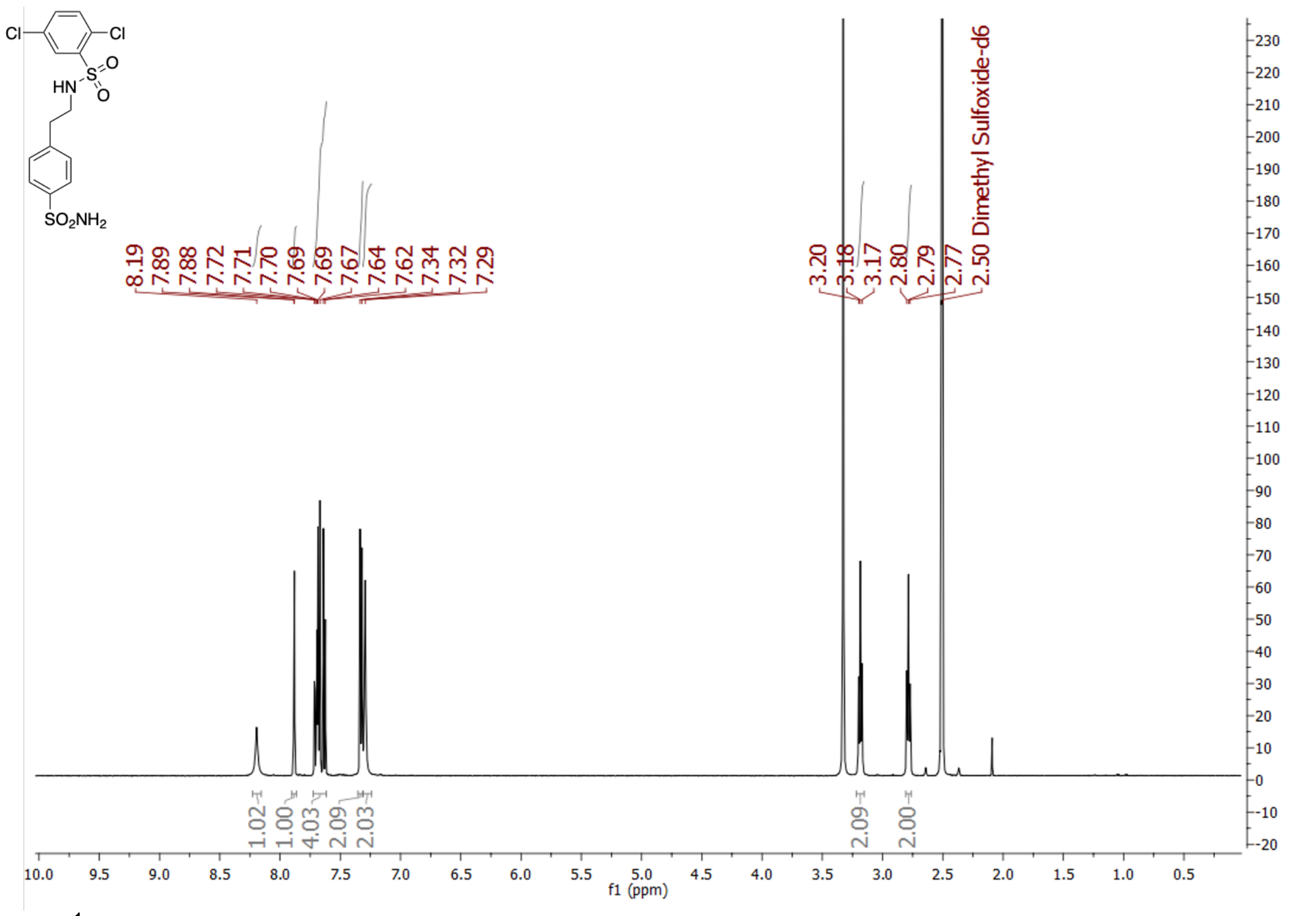

Fig. S12: ^{1}H NMR spectrum of 2,5-dichloro-*N*-(4-sulfamoylphenethyl)benzenesulfonamide (**16**)

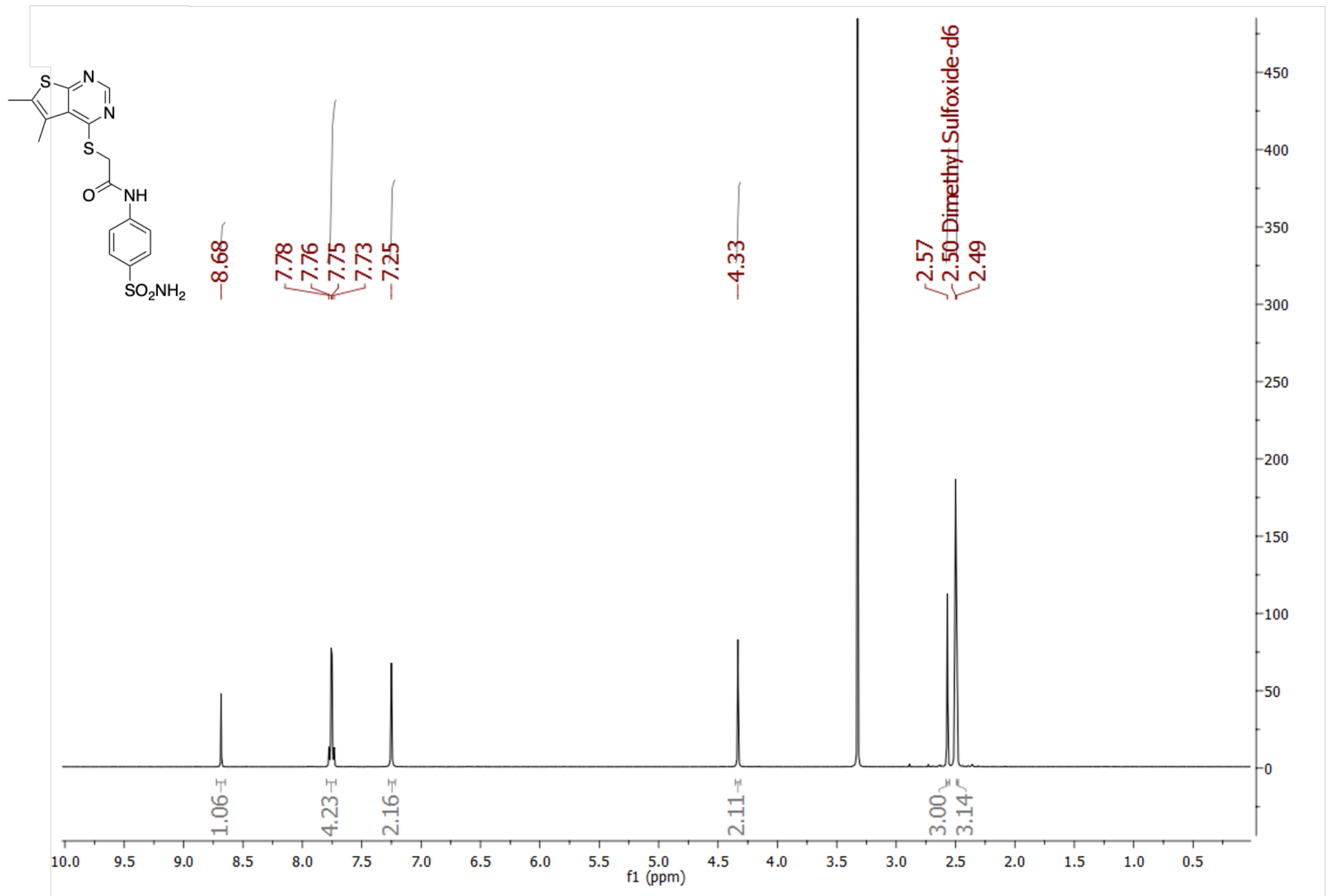

Fig. S13: ^{1}H NMR spectrum of 2-((5,6-dimethylthieno[2,3-*d*]pyrimidin-4-yl)thio)-*N*-(4-sulfamoylphenyl)acetamide (**17**)

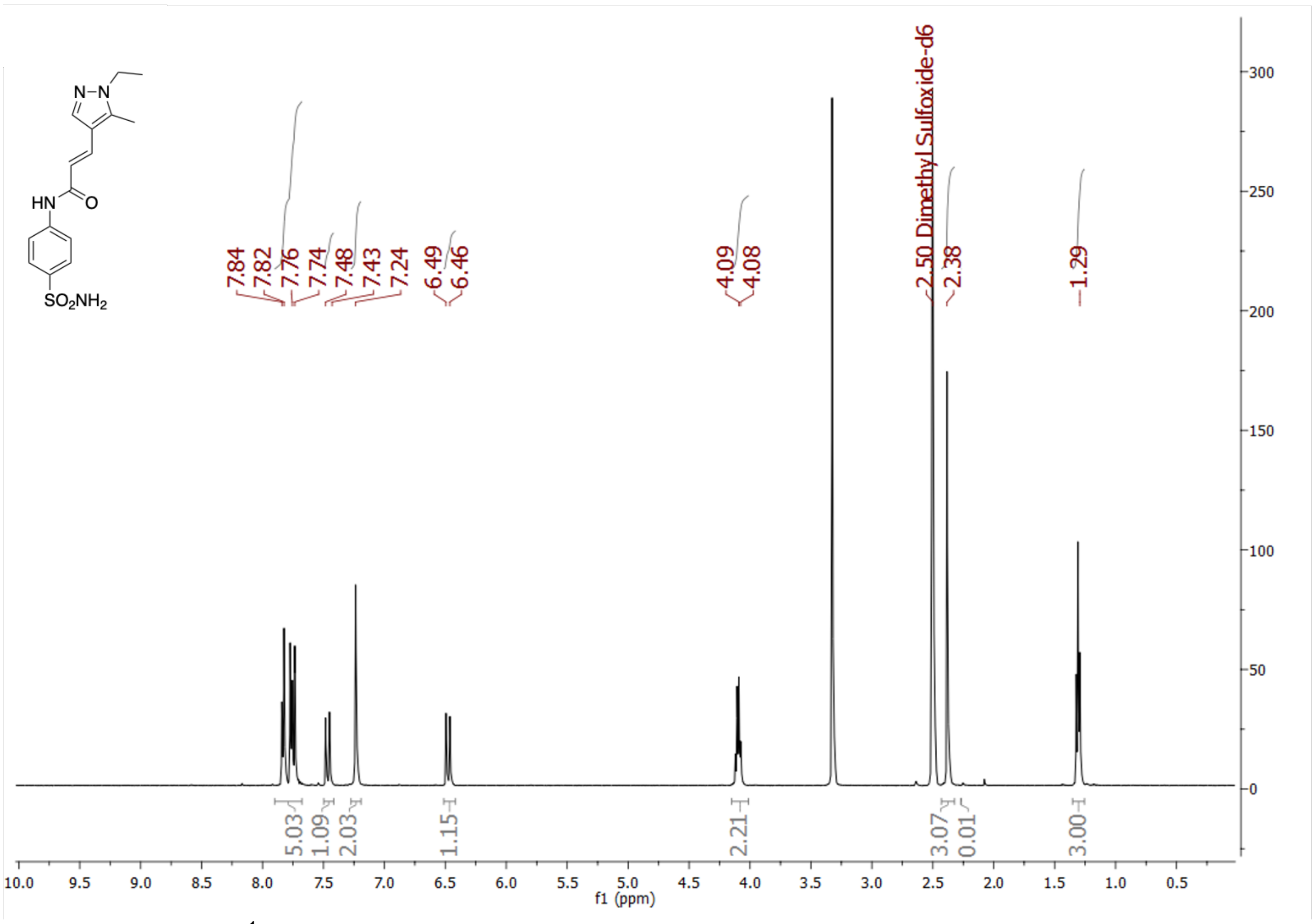

Fig. S14: ^{1}H NMR spectrum of 3-(1-ethyl-5-methyl-1*H*-pyrazol-4-yl)-*N*-(4-sulfamoylphenyl)acrylamide (**19**)

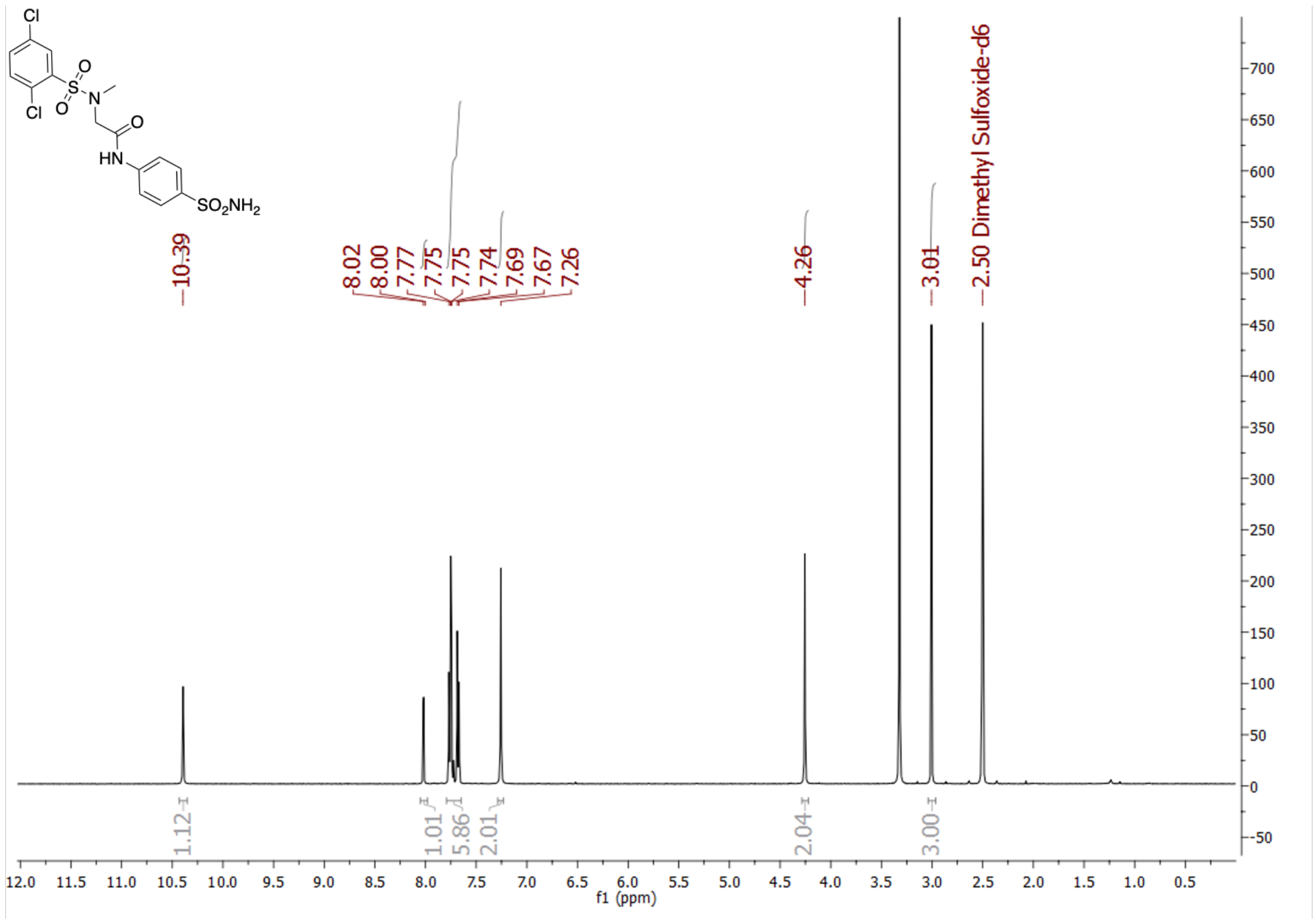

Fig. S15: ^{1}H NMR spectrum of 2-((2,5-dichloro-*N*-methylphenyl)sulfonamido)-*N*-(4-sulfamoylphenyl)acetamide (**20**)

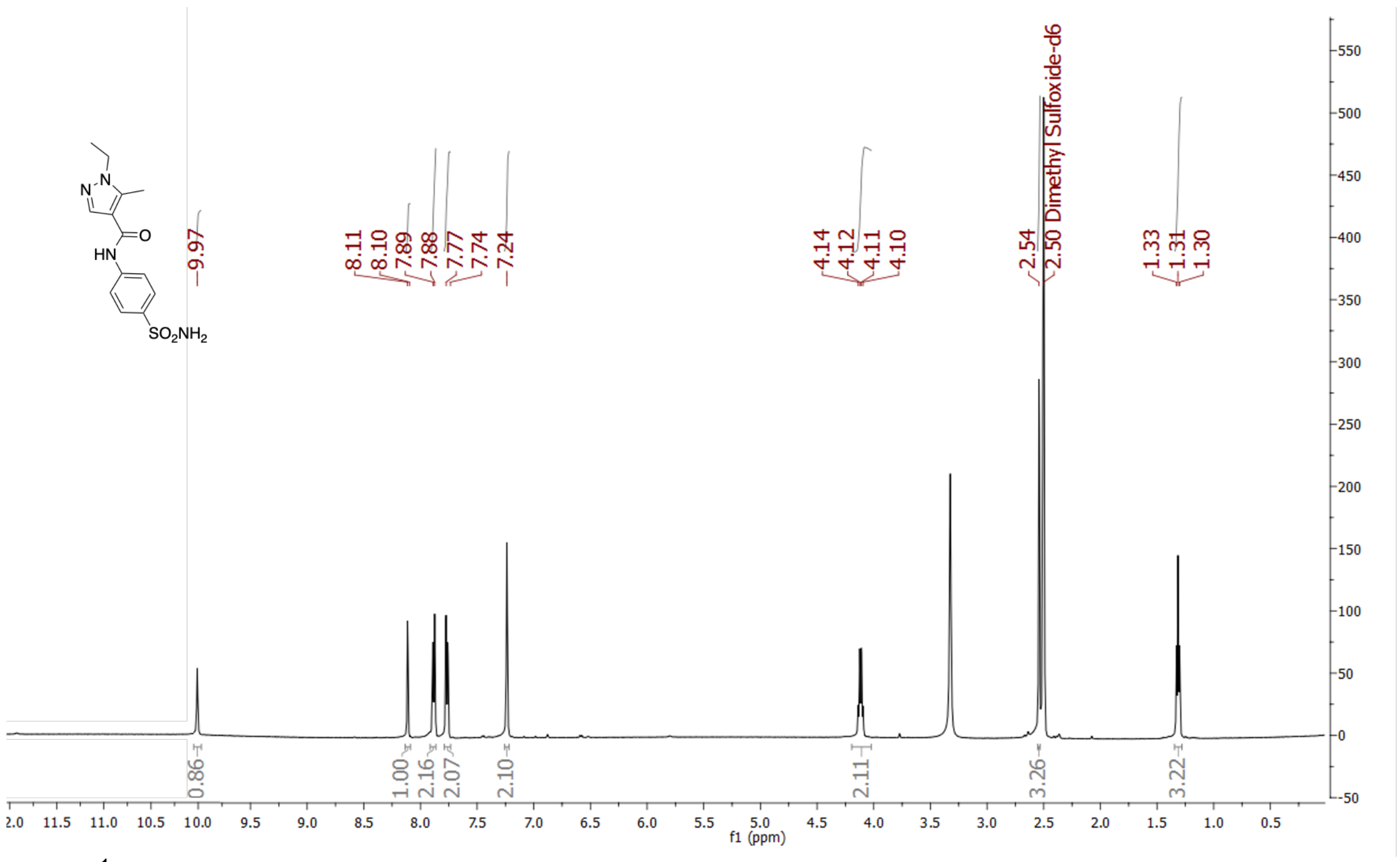

Fig. S16: ^{1}H NMR spectrum of 1-ethyl-5-methyl-*N*-(4-sulfamoylphenyl)-1*H*-pyrazole-4-carboxamide (**22**)

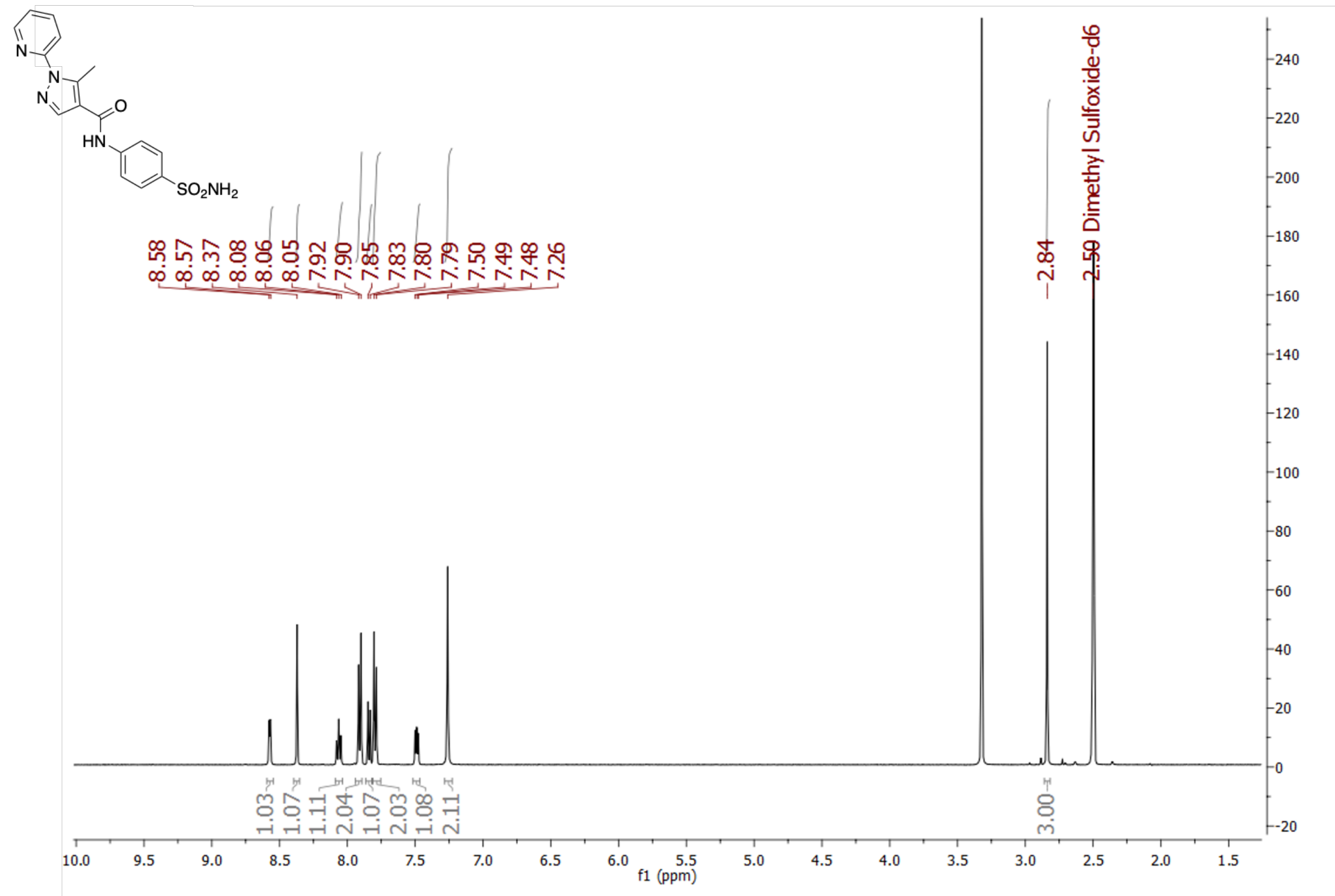

Fig. S17: ^{1}H NMR spectrum of 5-methyl-1-(pyridin-2-yl)-*N*-(4-sulfamoylphenyl)-1*H*-pyrazole-4-carboxamide (**24**)

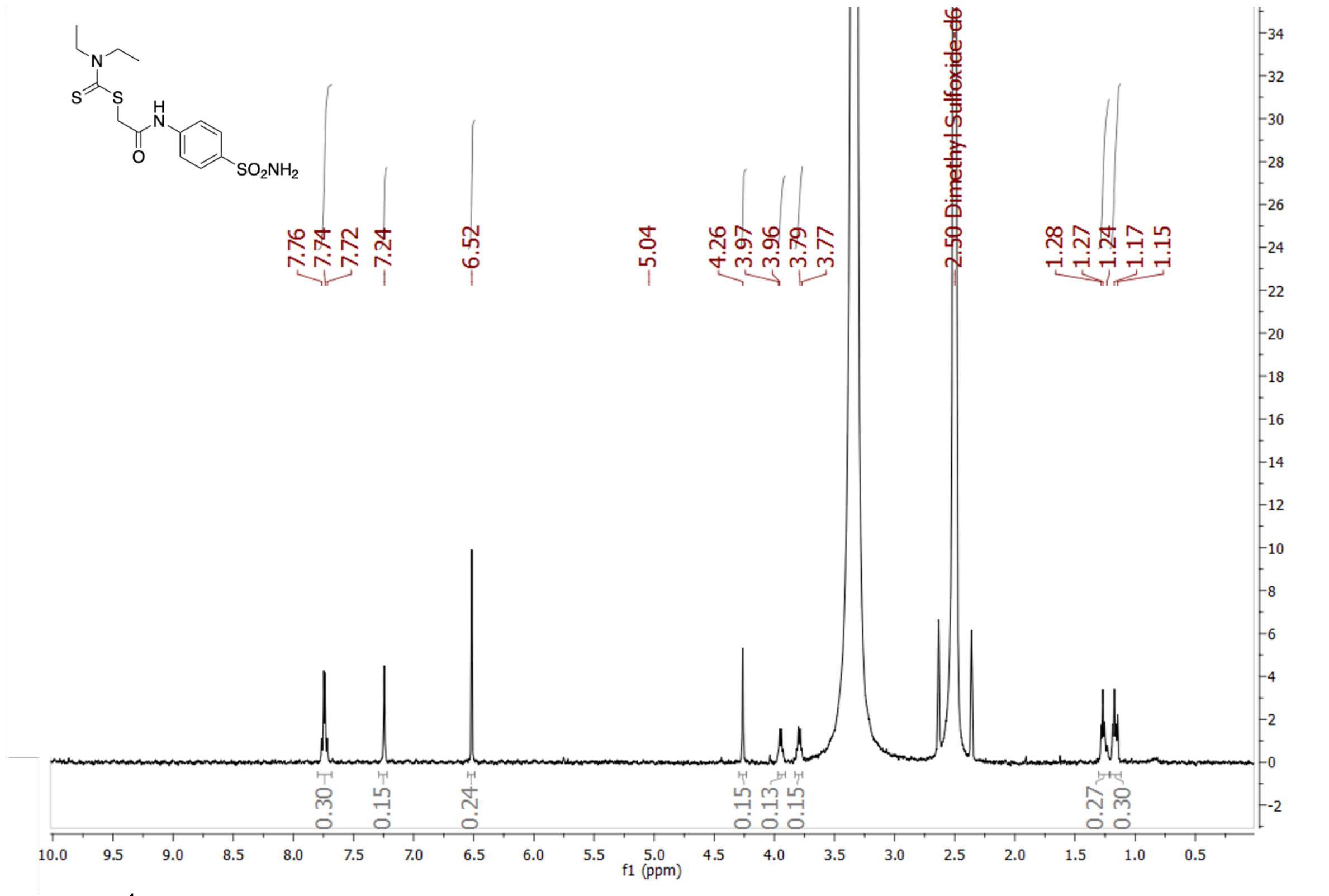

Fig. S18: ^{1}H NMR spectrum of 2-oxo-2-((4-sulfamoylphenyl)amino)ethyl diethylcarbamodithioate (**25**)

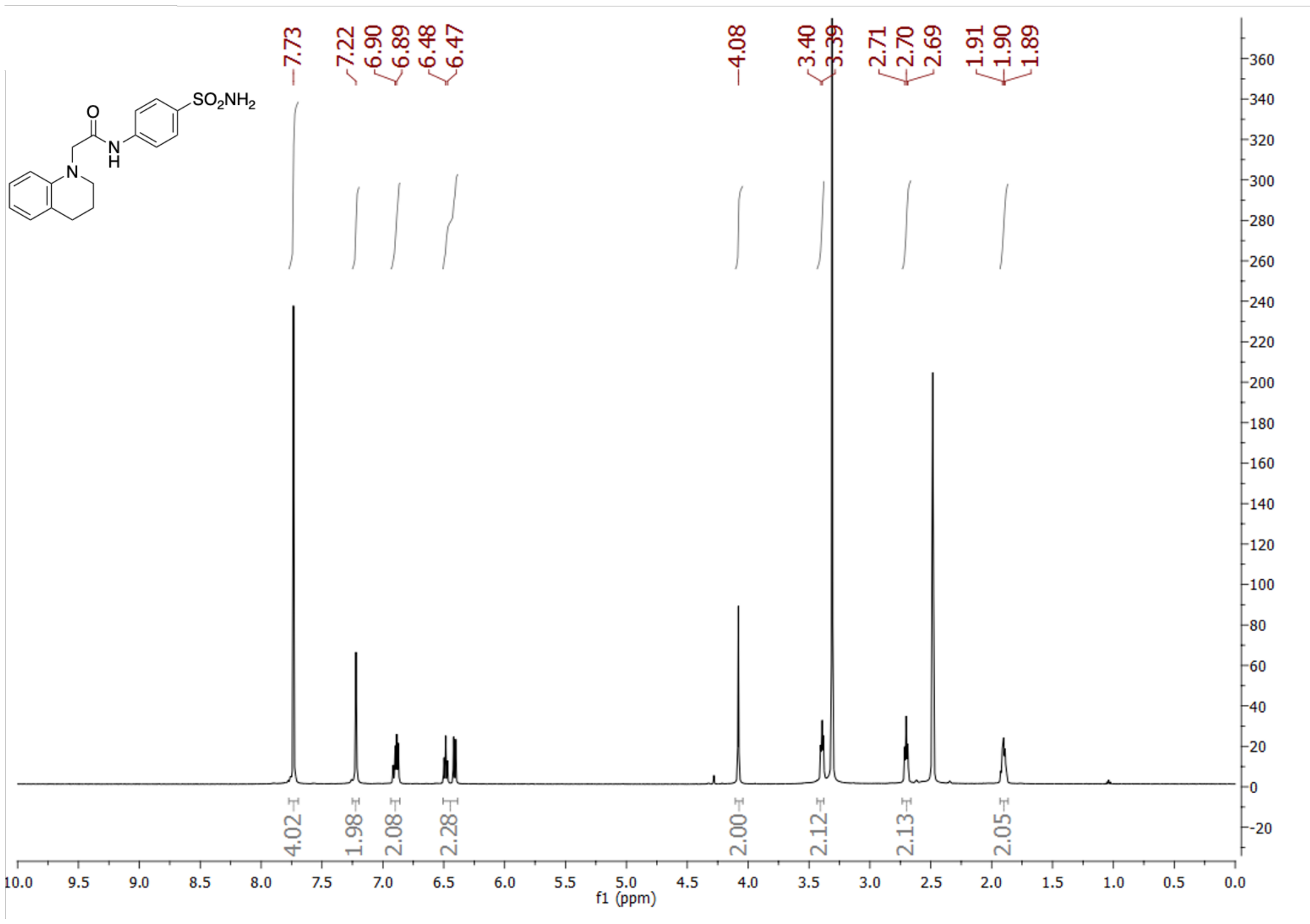

Fig. S19: ^{1}H NMR spectrum of 2-(3,4-dihydroquinolin-1(2*H*)-yl)-*N*-(4-sulfamoylphenyl)acetamide (**26**)

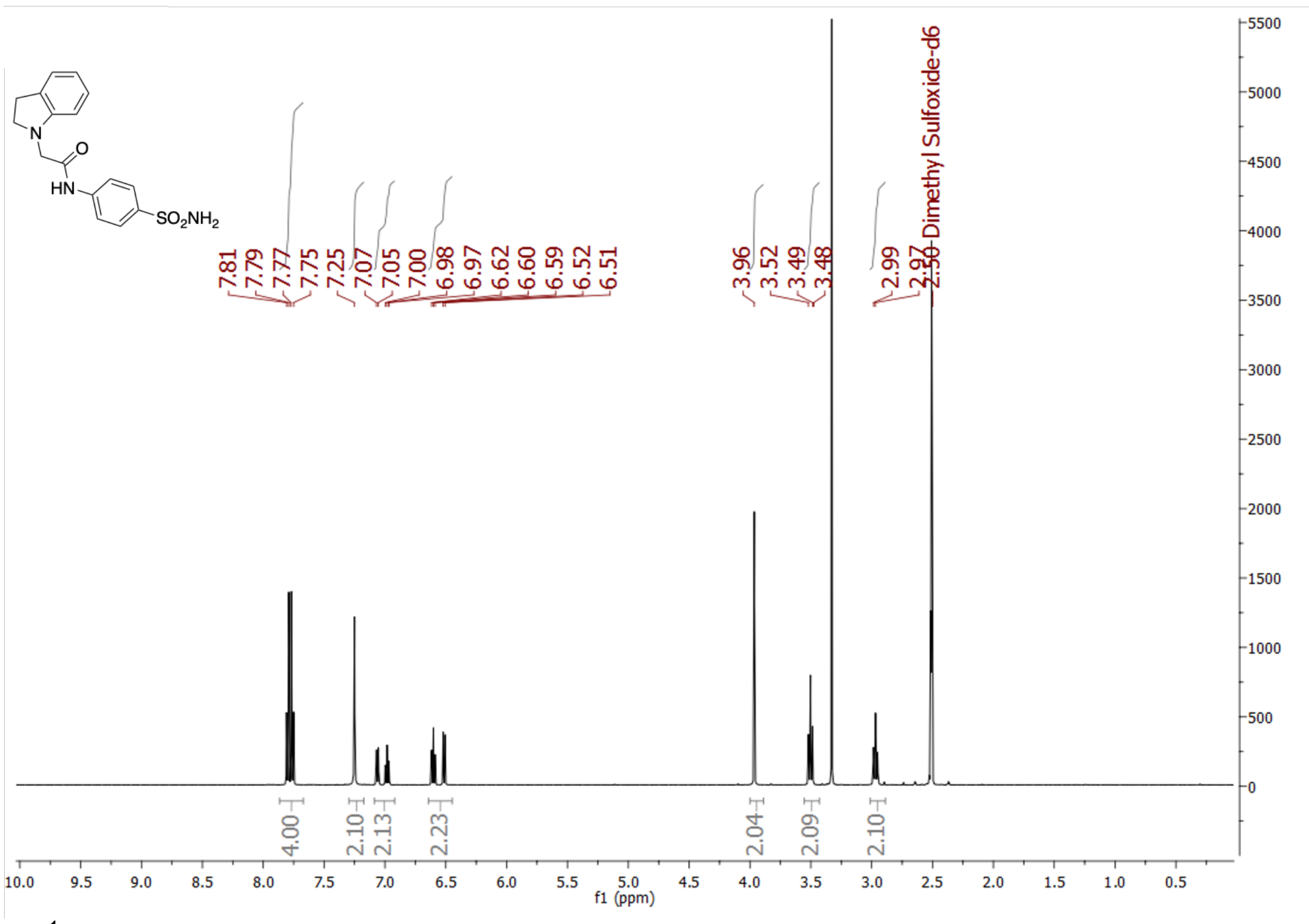

Fig. S20: ^{1}H NMR spectrum of 2-(indolin-1-yl)-*N*-(4-sulfamoylphenyl)acetamide (**27**)
